# Experimental trials of predicted CD4^+^ and CD8^+^ T-cell epitopes of respiratory syncytial virus

**DOI:** 10.3389/fimmu.2024.1349749

**Published:** 2024-04-02

**Authors:** Syeda Tahira Qousain Naqvi, Syed Aun Muhammad, Jinlei Guo, Sidra Zafar, Amjad Ali, Larry J. Anderson, Christina A. Rostad, Baogang Bai

**Affiliations:** ^1^ Institute of Molecular Biology and Biotechnology, Bahauddin Zakariya University, Multan, Pakistan; ^2^ School of Intelligent Medical Engineering, Sanquan College of Xinxiang Medical University, Xinxiang, Henan, China; ^3^ Atta-ur-Rehman School of Applied Biosciences (ASAB), National University of Sciences and Technology (NUST), Islamabad, Pakistan; ^4^ Department of Pediatrics and Children’s Healthcare of Atlanta, Emory University, Atlanta, GA, United States; ^5^ School of Information and Technology, Wenzhou Business College, Wenzhou, Zhejiang, China; ^6^ Engineering Research Center of Intelligent Medicine, Wenzhou, Zhejiang Province, China; ^7^ The First School of Medical, School of Information and Engineering, The First Affiliated Hospital of Wenzhou Medical University, Wenzhou, Zhejiang, China

**Keywords:** RSV, prophylactic vaccine, healthy and diseased animal models, hematological and cellular assays, virus neutralization assay

## Abstract

**Background:**

Respiratory syncytial virus (RSV) is the most common cause of viral lower respiratory tract infections (LRTIs) in young children around the world and an important cause of LRTI in the elderly. The available treatments and FDA-approved vaccines for RSV only lessen the severity of the infection and are recommended for infants and elderly people.

**Methods:**

We focused on developing a broad-spectrum vaccine that activates the immune system to directly combat RSV. The objective of this study is to identify CD4^+^ and CD8^+^ T-cell epitopes using an immunoinformatics approach to develop RSV vaccines. The efficacy of these peptides was validated through *in-vitro* and *in-vivo* studies involving healthy and diseased animal models.

**Results:**

For each major histocompatibility complex (MHC) class-I and II, we found three epitopes of RSV proteins including F, G, and SH with an antigenic score of >0.5 and a projected SVM score of <5. Experimental validation of these peptides on female BALB/c mice was conducted before and after infection with the RSV A2 line 19f. We found that the 3RVMHCI (CD8^+^) epitope of the F protein showed significant results of white blood cells (19.72 × 10^3^ cells/μl), neutrophils (6.01 × 10^3^ cells/μl), lymphocytes (12.98 × 10^3^ cells/μl), IgG antibodies (36.9 µg/ml), IFN-γ (86.96 ng/L), and granzyme B (691.35 pg/ml) compared to control at the second booster dose of 10 µg. Similarly, 4RVMHCII (CD4^+^) of the F protein substantially induced white blood cells (27.08 × 10^3^ cells/μl), neutrophils (6.58 × 10^3^ cells/μl), lymphocytes (16.64 × 10^3^ cells/μl), IgG antibodies (46.13 µg/ml), IFN-γ (96.45 ng/L), and granzyme B (675.09 pg/ml). *In-vitro* studies showed that 4RVMHCII produced a significant level of antibodies in sera on day 45 comparable to mice infected with the virus. 4RVMHCII also induced high IFN-γ and IL-2 secretions on the fourth day of the challenge compared to the preinfectional stage.

**Conclusion:**

In conclusion, epitopes of the F protein showed considerable immune response and are suitable for further validation.

## 1 Introduction

Respiratory syncytial virus (RSV), a highly infectious agent and a major cause of lower respiratory tract infections (LRTIs), was discovered in 1955 (1). RSV has 10 open reading frames (ORFs) in its genome, which encode 11 structural and non-structural proteins. The initial seven genes produce seven structural proteins. Viral RNA is encapsulated by the nucleoprotein (N), phosphoprotein (P), and RNA-dependent RNA polymerase (L), resulting in a helical assembly known as the ribonucleoprotein complex (RNP). This structure forms the minimal replication machinery and protects viral RNA. The RSV contains three important membrane proteins: the short hydrophobic protein (SH), the attachment glycoprotein (G), and the fusion protein (F). The G protein is involved in viral attachment and the F protein helps in viral fusion with host cells. A pentameric ion channel is formed by the SH protein (2). Infants (<2 years), elderly people (>65 years), and cardiovascular or lung disease or immunosuppressed individuals are more susceptible to RSV infections. RSV infection may reoccur in individuals at any stage of their life. In young and healthy individuals, this infection is usually mild but, in older adults and infants, it can lead to major consequences (3). Approximately, 30 million people are infected annually and 3 million people are hospitalized with RSV infections. Currently, after the COVID-19 epidemic, the rate of RSV infection and hospitalization has increased significantly not only in the USA but also in other 20 geographical regions (4). In general, annual deaths from RSV-related LRI may be between 55,000 and 200,000 (5). In Pakistan, a low-income country, RSV is considered a major source of LRI and mortality of < 5-year-old children. RSV-associated respiratory diseases account for 20%–30% of all pediatric mortalities (6). Since there is no specific treatment for RSV infections, their prevalence and impact on health are substantial. As supportive treatments, currently available medications including palivizumab and ribavirin only work to reduce the intensity and symptoms of infection (7). There are some difficulties reported for RSV vaccine development, for example, infant age, chances of reoccurrence of infection associated with formalin-inactivated vaccine (FI), and the hypersensitivity issues linked with subunit vaccine (8). An efficient vaccine should be able to produce neutralizing antibodies, inhibit viral replication, and stop the development of infections. The tragic reality is that no effective and successful vaccination was approved for use until early 2023 (9). In the mid of 2023, the FDA approved two new vaccines including Arexvy and Abrysvo with some limitations and side effects, as they only target specific age groups (10, 11).

Historically, vaccines for diseases were developed empirically by isolating, killing, or inactivating, and then administering the pathogens (complete or part of them) responsible for the disease (12). The other scenario involved the purification of proteins before using them as potential vaccines (13). More than 20 years ago, genome sequencing transformed these traditional procedures by allowing the direct use of genetic data for the development of novel vaccine antigenic candidates. The importance of this research is to develop a vaccine that will specifically stimulate the host immune system against RSV infection. By doing so, it will be possible to prevent RSV diseases and reduce its infectious burden. In addition, vaccines against additional viral diseases can be developed following the procedures used in this research. In this study, we used the reverse vaccinology (RV) approach to predict and design potential vaccine candidates based on a system-level framework (12). This approach is based on computational techniques that seek potential vaccine candidates using the pathogen proteomic data. The recently reported subunit vaccine of COVID-19 (14) has been designed using this approach. Such techniques are also being applied to combat HIV and influenza infections (12). Emerging techniques in human structural biology and immunology offer new molecular data to predict and design vaccines against human CMV (human cytomegalovirus) and RSV. The prediction of T-cell and B-cell epitopes is one aspect of this approach. Improvements in epitope prediction tools have reduced the amount of expensive and time-consuming screening required in the past. Recently developed *in-silico* prediction techniques significantly reduce the workload related to epitope mapping by reducing the number of probable epitope candidates that must be tested experimentally (15). Vaccines containing B-cell epitopes that trigger a humoral immune response may provide short-term immunity, while T-cell vaccines may trigger long-term cell-mediated immunity with minute humoral immune responses (16). Understanding the distinction between “self” and “non-self” is essential for predicting T-cell epitopes, which are the parts of pathogens recognized by immune cells. This knowledge plays a crucial role in the fight against infections and the prevention of autoimmune diseases. In the case of RSV infections, where there is currently no specific treatment available, it becomes even more vital to understand how T-cell epitopes interact with host molecules. It is worth noting that the immune system’s mechanism of “negative selection” ensures that T cells reacting to self-peptides are eliminated, thus maintaining tolerance toward our tissues (17). When epitopes are presented by MHC molecules, T lymphocytes can identify them. T cells experience positive selection early in the thymic growth phase to ensure that they bind to host MHC molecules. There are two types of MHC molecules: class I molecules appear on the surface of all nucleated cells, but class II molecules are only present on the surface of particular antigen-presenting cells (APCs) (18). Two different subsets of T cell are present due to the presence of two distinct classes of MHC molecules: CD4^+^ and CD8^+^ T cells specifically bind to MHCI and MHCII, respectively (17). Experimental validation of predicted epitopes is a very crucial step in vaccine design. Animal models are used to validate vaccine candidates at various stages of vaccine development (19, 20).

The main objective of this research was to employ computational techniques to predict the T-cell epitopes of the F, G, and SH proteins of RSV. These peptides were synthesized and experimentally analyzed e to determine their efficacy as potential vaccine candidates. The successful development of RSV vaccines based on T-cell epitopes could provide a groundbreaking and focused strategy to combat RSV disease. In addition, if this strategy works, it may be a useful paradigm for vaccine development against other viral infections.

## 2 Materials and methods

### 2.1 Ethical approval

The Institute of Molecular Biology and Biotechnology Animal Bioethics Committee approved the ethics of animal studies under Approval No. IMBB/02/2019, and Emory University’s Institutional Animal Care and Use Committee approved for phase II (Atlanta GA, USA). Animal studies were conducted on healthy and diseased models (infected with the RSV A2l19f strain obtained from Anderson’s Laboratory in Children’s Healthcare of Atlanta, Emory University, Atlanta, GA, USA). Our hypothesis interprets the activation of CD4^+^ and CD8^+^ lymphocytes by these potential and T-cell-specific epitopes followed by the production of effector molecules. The systematic and integrative framework of this study is presented in **Supplementary Figure S1**.

### 2.2 Retrieval and screening of RSV proteomic data

RSV strain type A was selected based on clade-specific pathogenicity and significantly associated pathogenesis in newborns (21, 22). Protein sequences, in FASTA format for the RSV strain type A, were retrieved from the NCBI and Uniprot databases. These proteins were further screened based on molecular weight, antigenicity (threshold level 0.45), and subcellular localization to predict the T-cell epitopes (23). The protein-to-protein interaction network of antigenic RSV proteins with other host proteins was generated by Cytoscape version 3.6 (24).

### 2.3 Prediction and screening of T-cell epitope

ProPred-I and ProPred servers were used to predict the multi-allelic CD8^+^ and CD4^+^ T-cell epitopes related to MHCI and MHCII, respectively (25). The predicted epitopes were selected based on antigenicity using VaxiJen v2.0 (threshold value 0.5), immunogenicity using the IEDB server, toxicity through ToxinPred (SVM score <5), binding affinity (<−0.5 KJ/mol) with MHC class I and II targets using MOE (Molecular Operating Environment software ver. 2014.09), and the physicochemical properties of epitopes through Expasy ProtParam as reported in our study (23).

### 2.4 Conservation analysis of predicted epitopes

To explore the broad spectrum of global effects, we examined the conservation of the predicted epitopes. Using the IEDB tool, this conservation investigation was conducted on various RSV strains associated with human diseases (26). The phylogenetic tree was also constructed to study the relationship among the above-mentioned strains using MEGA 11 (27). To reduce the risks and negative impacts of putative antigenic epitopes, non-human homologous proteins were found using BLAST analysis on the NCBI database against humans (Homo sapiens).

### 2.5 Synthesis of peptides

The predicted T-cell epitopes of MHCI (CD8^+^) and MHCII (CD4^+^) were synthesized from Shanghai Royobiotech Co., LTD, China (invoice #2019NY0327-ZN9068).

### 2.6 Dose optimization

For *in-vivo* studies of predicted T-cell peptides to immunize mice, the dose was optimized following the dose scheme (**Table 1**). Six groups of mice (one for each peptide) were further classified into four subgroups labeled as control, independent peptide, peptide plus adjuvant, and only adjuvant. Alhydrogel was used as an adjuvant in our study. Synthesized peptides were administered in equal concentrations to the peptide group and peptide with the adjuvant groups. According to preclinical trial guidelines, the initial dose was evaluated and gradually increased until it was considered unsafe. The concentrations of 5 µg, 10 µg, and 15 µg were injected to mice every 3 weeks. The benchmark of the optimum dose used throughout the experiment was an increase in antigenic response. For the optimization process, the 4RV1 peptide was used. Blood was drawn to perform hematological and immunoassays 3 weeks after each dose.

**Table 1:** Plan for using synthetic peptides to immunize mice.

Epitope	Groups	Mice/group	Priming dose	Interval	First booster dose	Interval	Second booster dose
RV1/RV2/RV3/4RV1/5RV2/6RV3	Control	6 mice	50 μl PBS	3 weeks	50 μl PBS	3 weeks	50 μl PBS
	Peptide		5 μg/50 μl		10 μg/50 μl		10 μg/50 μl
	Peptide ^+^ adjuvant (alhydrogel)		25 µl alhydrogel + 5 µg + saline = 50 µl		25 µl alhydrogel + 10 µg + saline = 50 µl		25 µl alhydrogel + 10 µg + saline = 50 µl
	Adjuvant		50 μl adjuvant		50 μl adjuvant		50 μl adjuvant

### 2.7 Immunization of mice model

The female BALB/c mice of about 6 weeks with weight of 18–20 g were used for the experimental investigation of designed peptides (28). Mice were immunized subcutaneously using a 1ml syringe (29) and maintained in carefully regulated lab settings (20°C–22°C, 50%–60% humidity, 12h light cycle). A primary dose of 5 µg/50 µl of 1× PBS (phosphate buffer saline; pH 7.4) was injected into the independent peptide group, while 5 µg/25 µl PBS along with 25 µl of alhydrogel in the peptide plus adjuvant group. A similar scheme was applied for the first and second booster doses for the experimental and control groups. After immunization, animals were regularly monitored to observe their response (30).

### 2.8 Collection of blood samples

Using hematocrit capillaries with an inner diameter of 0.8 mm and a length of 75 mm, blood was extracted from immunized mice under carefully monitored conditions by rupturing their retro-orbital veins after 3 weeks of administration of priming, first, and second booster doses, respectively. The serum was separated by centrifugation at 2000*g* for 10 min at 4°C (29).

### 2.9 Hematological assays

The CBC Coulter (Convergys X3 NG, Germany; Model:1100-2600) was used to count lymphocytes, white blood cells (WBCs), and other components of blood samples of immunized mice, and the results were interpreted in 10^3^ cells/µl (31, 32).

### 2.10 Immunoassays

#### 2.10.1 IgG ELISA assays

The significance of IgG antibodies lies in their high specificity and long-lasting presence. These antibodies serve as valuable tools to identify previous infections and assess the efficacy of vaccines (33). The IgG ELISA of sera from immunized mice was performed at room temperature (RT) by following the manufacturing protocol of the Antigen Down ELISA Development Kit (Catalog # 9101). On the first day, the respective wells were coated with peptides (4 µg/ml) and kept overnight incubation. The plates were blocked by adding 100 µl/well block buffer after washing and then kept for overnight incubation. On the next day, we dried the wells by tapping on absorbent paper, added 100 µl of mouse serum samples to their respective wells, and incubated for 45 min. Washed the plate, 100 µl/well of HRP-conjugate was added and incubated for 1h. The color changed to blue-green after incubating 100 µl of TMB (3,3',5,5' tetramethylbenzidine) substrate per well for 15–20 min in the dark. The IgG concentration in the samples (mg/ml) was estimated by measuring the absorbance at 450 nm after adding 100 µl/well of stop solution, which caused the observed color to turn yellow (34).

#### 2.10.2 IFN-γ ELISA assay

Interferon-gamma (IFN- γ) is an important effector molecule produced by the CD4^+^ T-lymphocytes in response to antigen exposure (35). To check the efficacy and response of these CD4^+^ T-cell-specific epitopes, we performed an IFN-γ assay using an IF3N-γ ELISA Kit (Catalog #E0056Mo) (36). Initially, all reagents were kept at room temperature and the assay was performed as per standard guidelines. The standard solution was serially diluted using standard diluent (1:2) from 640 to 40 ng/L. Fifty microliter of standard, 50 µl of diluent (blank), and 40 µl of serum samples were added to their respective wells. Ten microliter of IFN-γ-specific antibody was added to each sample well followed by the addition of 50 µl of Streptavidin-HRP to each well. Each sample was gently mixed and incubated at 37°C for 60 min. After washing, 50 µl of substrate solutions A and B were added and incubated at 37°C for 10 min in the dark. After adding 50 µl of stop solution, the absorption was measured at 450 nm using a Bio-Rad (xMark™ Microplate Absorbance Spectrophotometer; Model: 1681150) microplate reader after 10 min.

#### 2.10.3 Granzyme-B assay

The granzyme-B (GzmB) assay is a valuable tool in vaccine validation. Measuring T-cell-mediated cytotoxicity (CD8^+^) provides essential information about the vaccine’s efficacy beyond humoral immunity contributing to the design of safer and more effective vaccines for the future. The GzmB and perforin (Prf) were utilized by CD8^+^ T lymphocytes to eliminate tumor and virus-infected cells. Using the Mouse GzmB ELISA kit (Catalog #EM0420) manufacturer’s instructions, the amount of GzmB secreted in response to immunization with specific RSV peptides was measured in the serum of immunized animal models. One hundred microliter of each standard dilution (1: 2) from 100 to 1.56 pg/ml and sample serum (1:2) were added to their respective wells, and 100 µl of dilution buffer in the sample wells were added and kept for incubation at 37°C for 90 min. One hundred microliter of biotin-labeled antibody (one of 100) per well was added after washing, and the mixture was kept for incubation for 60 min at 37°C. After adding 100 µl of HRP-Streptavidin conjugate (one of 100), the mixture was incubated at 37°C for 30 min. Ninety microliter per well of TMB substrate was added to each well and incubated for 15–20 min. Fifty microliter of stop solution to each well was added and the absorbance of the wells was measured immediately at 450 nm using a BioTeck 800TS ELISA plate reader. The GzmB concentration was estimated and measured in pg/ml.

### 2.11 Disease model preparation: mice challenging with the A2l19f virus

Six peptides (one peptide per group, five animals per group) were injected intramuscularly into anesthetized BALB/c mice (6-week-old) after ketamine/xylazine (100 µl/mouse) IP injection. Normal saline was used to immunize the negative control group, while 1 × 10^6^ (TCID_50_) of the A2l19F virus was administered intraperitoneal to the positive control group using 100 µl of serum-free MEM media (minimal essential medium). The submandibular vein was used to draw blood in Eppendorf tubes on days 0 and 45 under isoflurane sedation and centrifuged at 8000*g* for 10 min for serum separation. The serum was stored at −20°C after being kept on ice throughout the procedure (37).

### 2.12 Neutralizing antibody assay

The serum viral neutralization test (SVN), used to determine the concentration and effectiveness of systemic antineutralizing antibodies that prevent a virus from infecting a host, is a highly accurate and focused technique (38). The neutralizing capacity of these antibodies was determined by twofold diluted sera from days 0 and 45 samples using the fluorescent focus unit (FFU) assay. Human epithelial type-2 cells (HEp-2) were seeded to produce a 70% confluency. On the day of the experiment, virus dilution [1 × 10^6^ FFU/ml (100 µl)] was prepared in MEM (900 µl) media. Sera samples were first diluted to 1:4 and then diluted twofold to 1:256 dilutions. Sixty microliter of virus dilution was added into 60 µl of diluted serum and incubated on the plate for 1h at 37°C. The media of the HEp2 wells was discarded and 50 µl of the virus/serum mixture was added to each well of the HEp2 plate. The plate was kept for 30 min at 4°C. After incubation, the plate was centrifuged at 2000 rpm followed by the addition of 150 µl of methylcellulose to each well and incubated for 2 days. Then the FFU/well was counted after the incubation period (39).

### 2.13 Stimulation of splenocytes with RSV CD4^+^- and CD8^+^-specific peptides

The spleen was harvested in cold R10 medium from immunized pre- and post-infected mice, euthanized with pentobarbital drug (200 µl), and homogenized splenocytes via a 70-µm cell strainer. 2 × 10^6^ cells (100 µl) of immunized mice were transferred to each well of a 96-well plate. Each sample had triplicate wells labeled as an unstimulated, leukocyte activation cocktail stimulated (LAC, 4 µl per 2 × 10^6^ cells), and peptide stimulated (20 µg/ml in R10) and incubated 1h at 37°C (35). In each unstimulated peptide well, 5 µl of Brefeldin A (GolgiPlug; 0.2 µl of Brefeldin A, 4.8 µl of R10) was added and incubated for 4 to 5h at 37°C. Centrifuged the plate for 3 min at 1200 rpm, resuspended the pellets into 38 µl of 2.4G2 Fc blocking mix (5 µl of 2.4G2 in 33 µl of FACS (fluorescence-activated cell sorting buffer), and incubated for 10 min on ice. 12.05 µl of the surface marker antibody cocktail (CD3 BUV496; 350 µl, CD4 BUV805; 43.8 µl, CD25 BV785; 70 µl, CD8a BB515; 43.8 µl, CD19 PerCP Cy5.5; 87.5 µl, CD11c PerCP Cy5.5;43.8 µl, CD11b PerCP Cy5.5; 116.9 µl, CD44 AF700; 43.8 µl, and IR Near L/D; 43.8 µl) was added to each well and kept in incubation for 15 min. The plate was centrifuged, the pellet broke down, and 75 µl of cold Cytofix buffer (1×) was added. Gently mixed buffer and 100 µl of cold permeabilization buffer (1×) were added to each well. The plate was centrifuged, and the pellet was resuspended into 50 µl of cytokine antibody (IL2 BV421: 140 µl, TNFα PE: 70 µl, IFNγ PE CF594: 70 µl, and 1× Permeabilization/Wash Buffer: 3220 µl) cocktail in permeabilization buffer. The plate was incubated overnight in the dark at 4°C. The next day, 100 µl of permeabilization buffer was added to each well and centrifuged at 1200 rpm for 3 min. The supernatant was removed by gently flicking the plate and vortexed the plate to break the pellets. Using FACS buffer, the pellet was resuspended and kept at 4°C in a dark room while flow cytometry was performed. Following four days of the challenge with 1 × 10^6^ FFU of mKate2-labeled A2-line19F, post-infection splenocytes were treated according to the same methodology. Run the flow to read the absorbance of samples at 300,000–500,000 events to observe the cellular response (40).

### 2.14 Evaluation of the lung viral load

We used the lung viral load assay to see the virus in the immunized mice’s lungs (41, 42). The left lung was removed after the four days of challenge and placed on ice in pre-labeled and pre-weighed labeled tubes. The lung was cut into three to four pieces into 400 µl of MEM medium and homogenized using the Mini-Bead-Beater with 1ml zirconia/silica beads (BioSpec Catalog #11079110z) for 1 min. This cycle was repeated 12 times and then centrifuged for 5 min at 2000 rpm. HEp2 cells (25,000 cells/well) were seeded in 96-well plates. Ninety microliter of NEAT (lung homogenate) was transferred in duplicate and 50 µl of virus A2l19f (1:10) per well was transferred to cell plates. The plates were centrifuged for 30 min at 2000 rpm at 4°C. Then, 150 µl of methylcellulose was added into each well. The plates were allowed to incubate for two days at 37°C before counting the FFU per well.

### 2.15 Statistical analyses

For statistical analysis, we used GraphPad Prism version 9, *t*- and *p*-values, standard deviations, standard error, and two-way analysis of variance (ANOVA) (43).

## 3 Results

### 3.1 Retrieved proteomic data

We retrieved a total of 11 proteins from the RSV type A isolate RSVNA1, as a reference genome from the NCBI and UniProt database. Of the 11 proteins, only three proteins were subcellular localized as transmembrane and extracellular proteins. The antigenicity study showed that these three transmembrane proteins; fusion (F), surface glycoprotein (G), and small hydrophobic (SH) were antigenic with an antigenic score of 0.57, 0.49, and 0.48, respectively (threshold value −0.45). These antigenic proteins were potential vaccine candidates because they possessed molecular weights of more than 7.5 kDa (44). The functional annotation of the screened proteins as a protein-protein interaction was given in the supplementary data (**Supplementary Figure S2**). The results of proteasomal cleavage of screened proteins were given in our previous study (23).

### 3.2 Predicted CD4^+^ and CD8^+^ T-cell epitopes

In our analysis using computational tools, we have identified 10 specific epitopes within the RSV proteins that could have the potential to bind specifically to CD4^+^ and CD8^+^ T cells, which in turn can trigger a robust antiviral immune response. Based on the antigenicity of the epitopes and their binding energies with the target alleles CD4^+^ and CD8^+^, such as HLA-A*01:01 for CD8^+^ (PDB ID: 1w72) and HLA-DRA/DRB1*01:01 for CD4^+^ (PDB ID: 1BX2), six epitopes were selected for further *in-vivo* experimentation (**Table 2**). Molecular interaction images along with binding energy values are provided as supplementary data (**Supplementary Figures S3**–**S5**) from our previously published paper (23). The physicochemical properties of the selected epitopes such as instability index, charge, toxin prediction, SVM score, hydropathicity, pI value, and aliphatic index were investigated (**Table 3**). CD8^+^ specific epitopes covered 17.34% of the world population with respect to target allele, while CD4^+^ specific epitopes covered 11.53%.

**Table 2:** Predicted T-cell epitopes for CD4^+^ and CD8^+^ as possible vaccination candidates.

Epitope sequence	Protein name	Peptide coding	Molecular weight (Da)	Antigenicity	SVM score	Toxicity	Binding energies
LKSIAQITL	G protein	1RVMHCI	986.22	0.6024	−1.18	Non-toxin	−8.5153
FSSKFWPYF	SH protein	2RVMHCI	1208.38	0.7148	−0.67	Non-toxin	−9.8595
LLALIAVGL	F protein	3RVMHCI	882.15	1.4491	−1.32	Non-toxin	−8.2264
IVRQQSYSI	F protein	4RVMHCII	1093.25	1.3637	−0.96	Non-toxin	−11.7086
LGISFSNLS	G protein	5RVMHCII	937.06	2.0701	−1.25	Non-toxin	−10.7463
FWPYFTLIH	SH protein	6RVMHCII	1223.44	1.2475	−1.12	Non-toxin	−11.5413

**Table 3:** The physicochemical characteristics of T-cell epitopes CD4^+^ and CD8^+^ as prospective vaccination candidates.

Peptide sequence	GRAVY	Charge	Half-life	Instability index (stable < 40)	Aliphatic index	pI value
LKSIAQITL	1.06	1.00	5.5h (*in-vitro* mammalian reticulocytes) 2 min (*in-vivo E. coli*) 3 min (*in-vivo* yeast)	−0.54	184.44	8.75
FSSKFWPYF	−0.100	1.00	1.1h (*in-vitro* mammalian reticulocytes) 2 min (*in-vivo E. coli*) 3 min (*in-vivo* yeast)	30.29	0.00	8.59
LLALIAVGL	3.01	0.00	5.5h (*in-vitro* mammalian reticulocytes) 2 min (*in-vivo E. coli*) 3 min (*in-vivo* yeast)	−0.54	271.11	5.52
IVRQQSYSI	−0.13	1.00	20h (*in-vitro* mammalian reticulocytes) >10h (*in-vivo E. coli*) 30 min (*in-vivo* yeast)	91.08	118.89	8.75
LGISFSNLS	0.96	0.00	5.5h (*in-vitro* mammalian reticulocytes) 2 min (*in-vivo E. coli*) 3 min (*in-vivo* yeast)	−0.54	130.00	5.52
FWPYFTLIH	0.69	0.50	1.1h (*in vitro* mammalian reticulocytes) 2 min (*in-vivo E. coli*) 3 min (*in-vivo* yeast)	22.6	86.67	6.74

### 3.3 Conservational analysis

**Figure 1:**
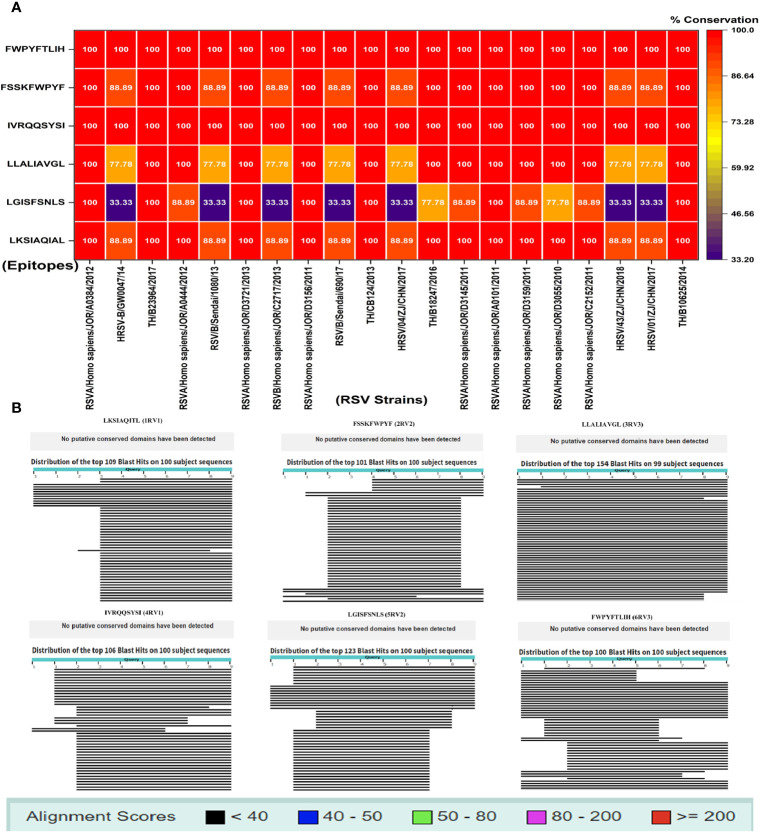
**(A)** Conservation study of putative vaccination epitopes using 20 pathogenic human RSV strains; **(B)** non-homologous sequence aliment of epitopes with human proteome.

Conservational analysis is important in vaccine design to evaluate the efficacy of these epitopes. In this analysis, two CD4^+^ T-cell epitopes including IVRQQSYSI and FWPYFTLIH demonstrated 100% conservancy (red) with different RSV strains and potentially have broad spectral effects. During conservation and sequence alignment, we found variations in the conservancy of CD4^+^ epitopes LGISFSNLS (blue) (33%–100%) while CD8^+^ T-cell epitopes including FSSKFWPYF, LKSIAQIAL, and LLALIAVGL exhibited more than 75% (orange) conservation (**Figure 1A**). Using human proteomic data as reference sequence, during sequence alignment, none of the six epitopes contain potential conserved domains (**Figure 1B**), indicating the dissimilarity of host and viral sequences. This step is important to avoid autoimmune responses. For evolutionary and phylogenetic analysis, we observed two main clades of RSV strains and found a 99%–100% evolutionary relationship and homology among various strains of RSV (**Supplementary Figure S6**).

### 3.4 Optimization of dose

**Figure 2:**
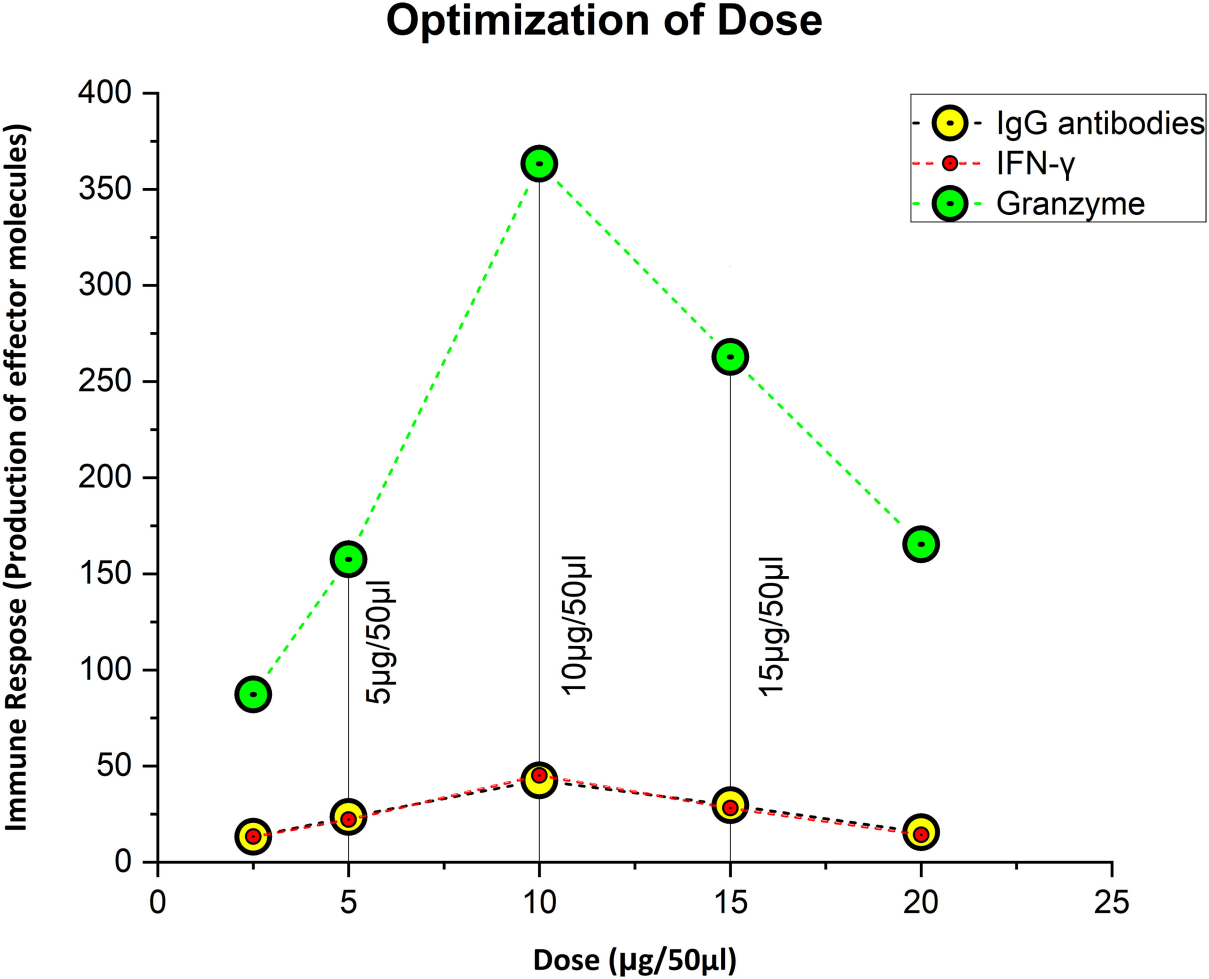
Figuring out the ideal, safe dosage. Ten microgram was thought to be the optimal dose, since it elicited the strongest immunological response, whereas 5 µg was thought to be the priming dose.

The dose was optimized by immunizing mice with concentrations of 5 µg, 10 µg, and 15 µg per 50 µl. As immunogenicity measures, IgG, IFN-γ, and GzmB concentrations were measured 3 weeks after the dose administration. In contrast to the control group, we observed that a dose of 5µg elicited an immunogenic response, whereas a dose of 10 µg greatly increased the level of stimulation of the immune system. At 15 µg, there was a reduction in IgG, IFN-γ, and GzmB concentration of considered a lethal dose. The mice showed stress-related symptoms and abnormal behavior at 15 µg, such as enlarged porphyrin rings, hair loss, frequent scratching, aggression, untidy coat, and less activity than usual, and 50% of the mice perished. So, 10 µg was the optimized dose as the first and second booster for the rest of the *in-vivo* studies (**Figure 2**). The immunological response of the third booster dose in an animal model was similar to that of the second booster dose. As a result, at this point we decided not to investigate into the third booster dose for further studies.

### 3.5 Hematological assays

**Figure 3:**
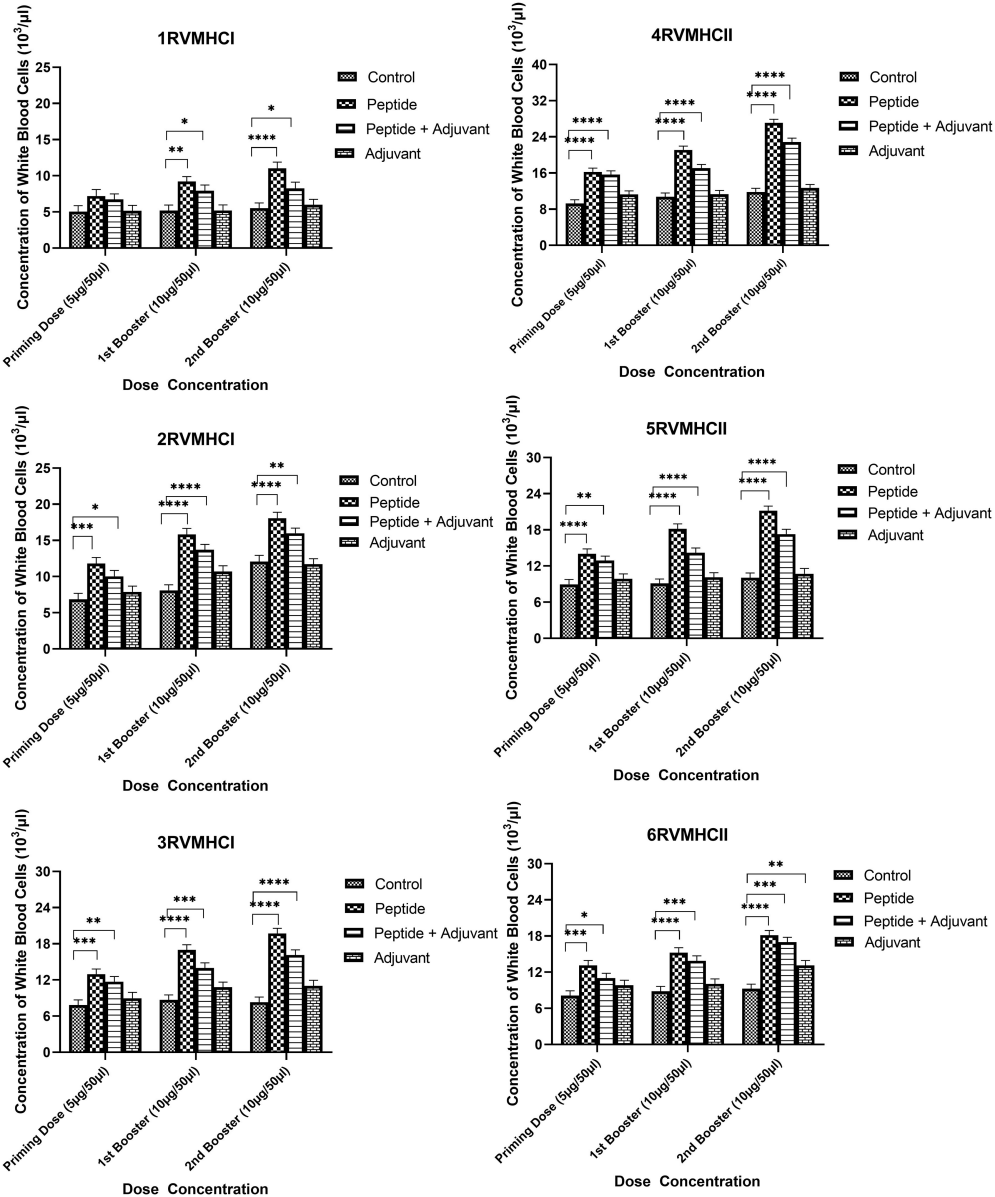
White blood cell counts in the sera of BALB/c mice given the different booster doses of CD4^+^ and CD8^+^ peptide immunization. The SEM from *n* = 6 mice/group was shown by error bars. Using GraphPad Prism version 9, two-way ANOVA and Dunnett’s multiple comparisons tests were used to establish the significant value (*p* < 0.05). (*p ≤ 0.05, **p < 0.01, ***p < 0.001, ****p < 0.0001).

**Figure 4:**
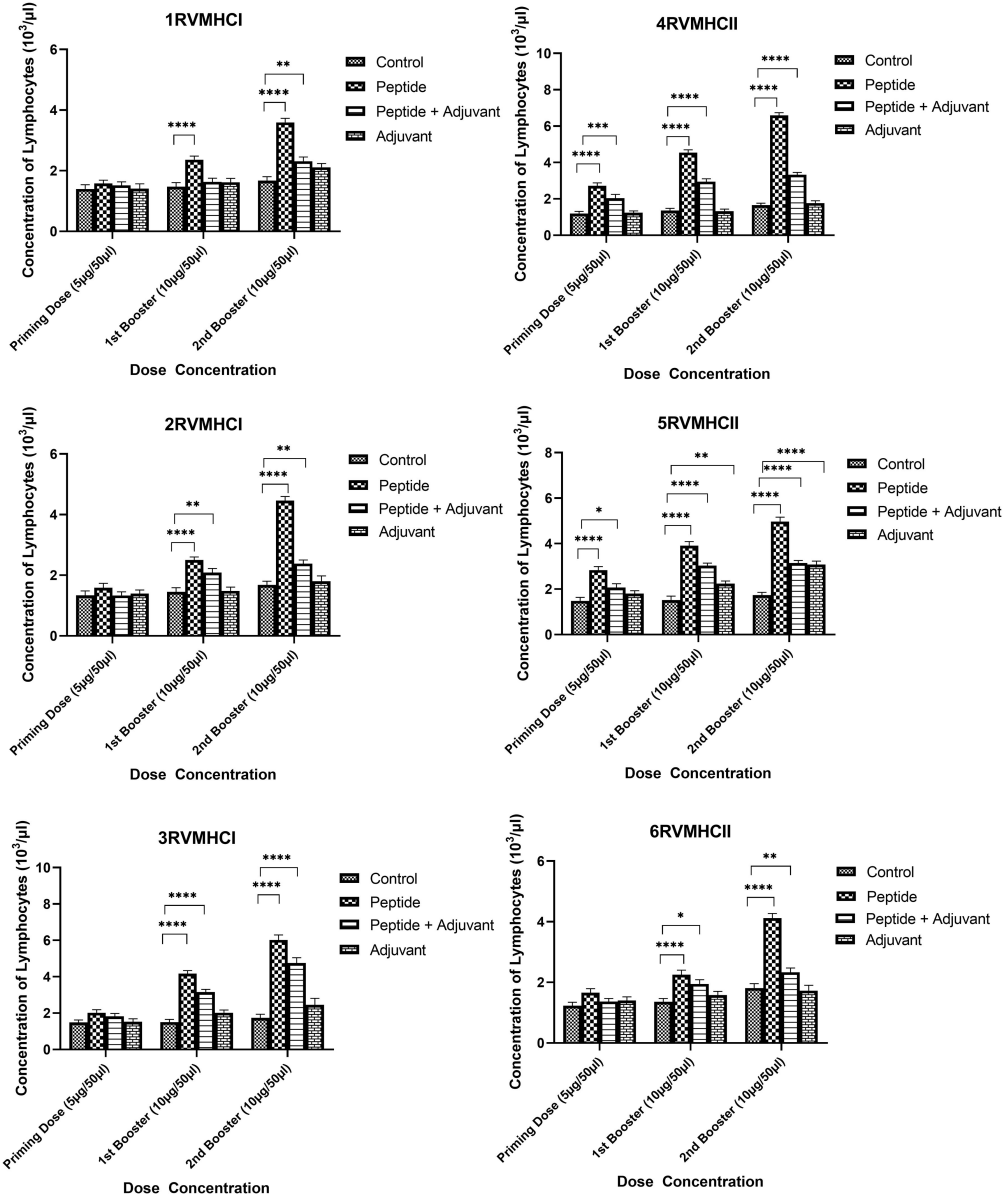
Lymphocyte counts in the sera of BALB/c mice given the different booster doses of CD4^+^ and CD8^+^ peptide immunization. The SEM from *n* = 6 mice/group was shown by error bars. Using GraphPad Prism version 9, two-way ANOVA and Dunnett’s multiple comparisons tests were used to establish the significant value (*p* < 0.05). (*p ≤ 0.05, **p < 0.01, ***p < 0.001,****p < 0.0001).

**Figure 5:**
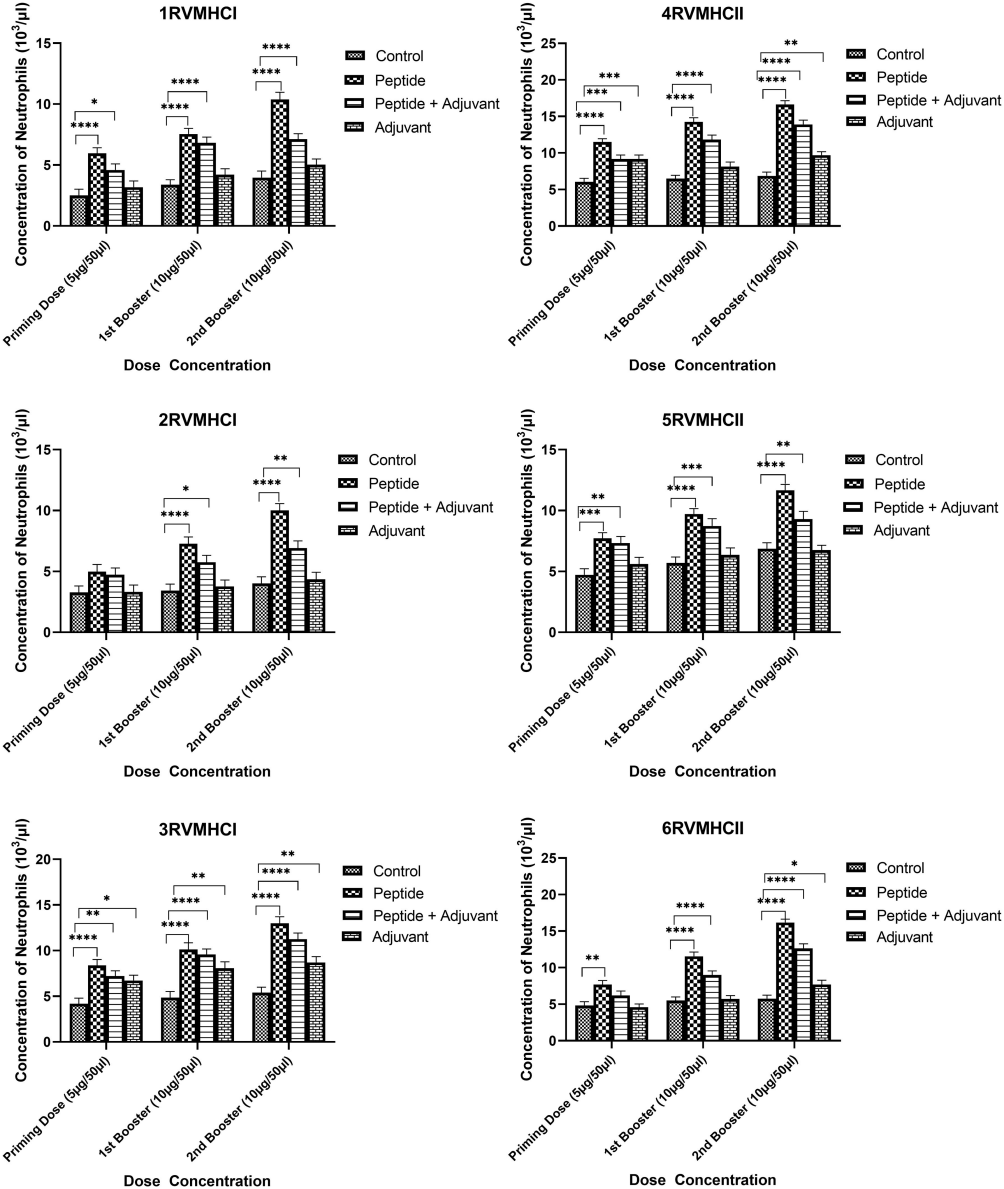
Neutrophil counts in the sera of BALB/c mice given the different booster doses of CD4^+^ and CD8^+^ peptide immunization. The SEM from *n* = 6 mice/group was shown by error bars. Using GraphPad Prism version 9, two-way ANOVA and Dunnett’s multiple comparisons tests were used to establish the significant value (*p* < 0.05). (*p ≤ 0.05, **p < 0.01, ***p < 0.001,****p < 0.0001).

The blood components, including WBC, LYM, and NEU, assessed in mice treated with peptides showed a significant immune response compared to their respective controls. The Dunnett’s multiple comparison test was used in a two-way ANOVA to compare the treatment groups of each peptide with the corresponding controls. According to this statistical study, each of the six distinct peptide groups had a significant number of blood components (*p*-value < 0.0001, 95% CI value) at 10 µg booster doses compared to all other groups (control, adjuvant, and peptide plus adjuvant). Analysis showed that of six epitopes, F protein CD8^+^ and CD4^+^ peptides (3RVMHCI and 4RVMHCII) independently observed relatively high levels of WBCs (19.72 × 10^3^ cells/μl and 27.08 × 10^3^ cells/μl, respectively), LYM (6.01 × 10^3^ cells/μl and 6.58 × 10^3^ cells/μl, respectively) and NEU (12.98 × 10^3^ cells/μl and 16.64 × 10^3^ cells/μl, respectively) in contrast to other groups at the 10 µg second booster dose and other immunization doses (**Figures 3**–**5**).

### 3.6 Immunoassays

#### 3.6.1 IgG immunoassays

**Figure 6:**
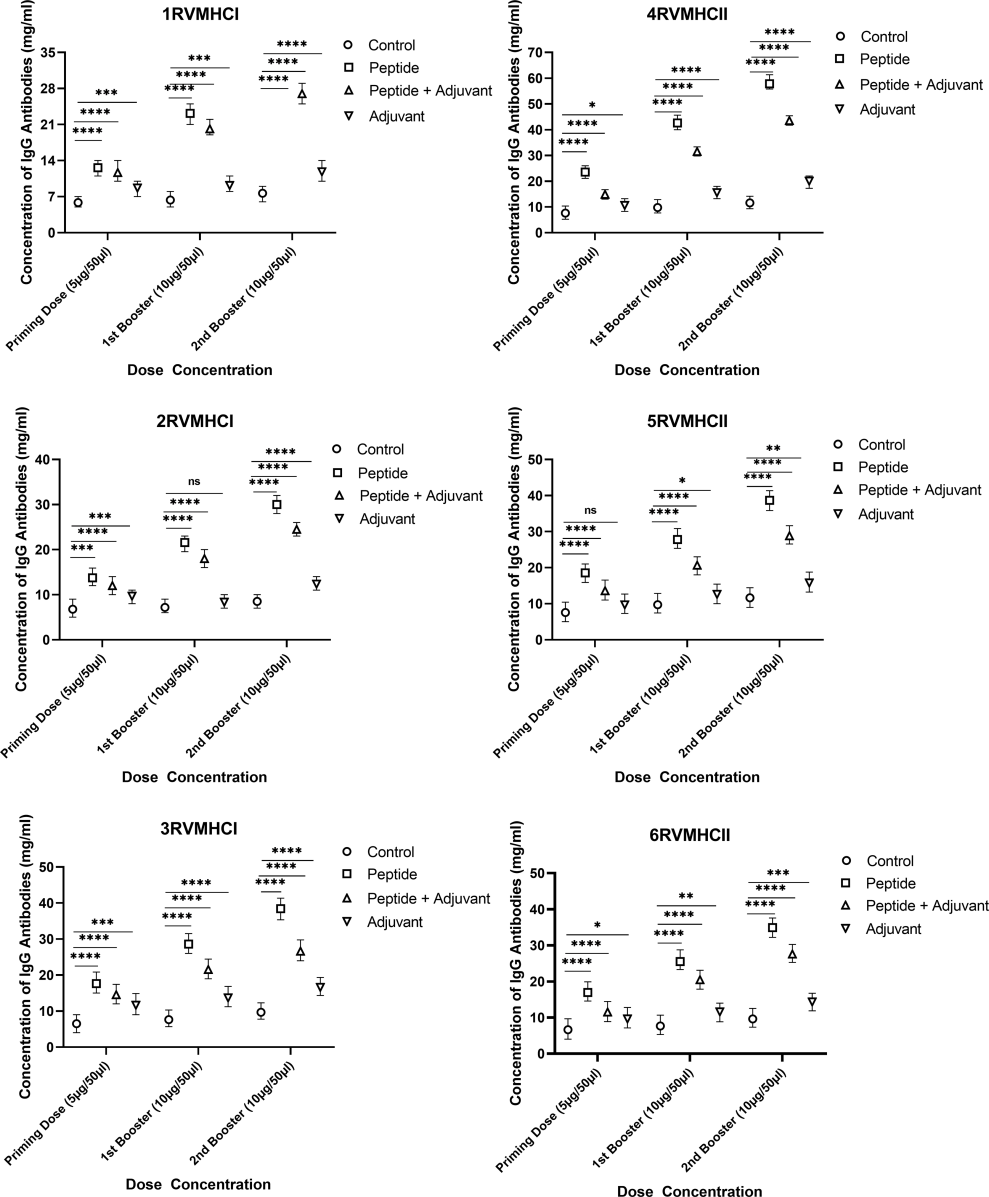
The levels of IgG antibodies (mg/ml) measured by ELISA, from sera of immunized BALB/c mice with different doses of synthetic peptides with and without adjuvant. The mean of six mice was represented by error bars. Using GraphPad Prism version 9, two-way ANOVA and Dunnett’s multiple comparisons tests revealed a significant difference (*P* < 0.05). (*p ≤ 0.05, **p < 0.01, ***p < 0.001,****p < 0.0001), ns: non-significant.

IgG ELISA is considered an essential assay for evaluating the humoral immune response of potential vaccine candidates. We found a substantial increase in IgG antibodies in independent peptide groups with a *p*-value of < 0.0001 compared to the peptides plus adjuvant and control groups in all doses, using two-way ANOVA with Dunnett’s multiple comparison test. Compared to priming doses, the second booster dose of all peptides significantly produced a high level of IgG antibodies. The CD8 + 3RVMHCI and CD4 + 4RVMHCII peptides of all of their respective peptides showed a significant increase in antibody concentration. A significant level of IgG antibodies (38.45 mg/ml) was observed in 3RVMHCI-treated mice independently at the second booster (10 µg) dose when compared to mice that showed 28.59 mg/ml and 17.64 mg/ml at the primary and first booster doses, respectively. Peptide 4RVMHCII also showed an induction trend of antibodies at 57.85 mg/ml, 42.60 mg/ml, and 23.51 mg/ml at the second, first booster, and priming doses, respectively (**Figure 6**).

#### 3.6.2 IFN-γ ELISA assay

**Figure 7:**
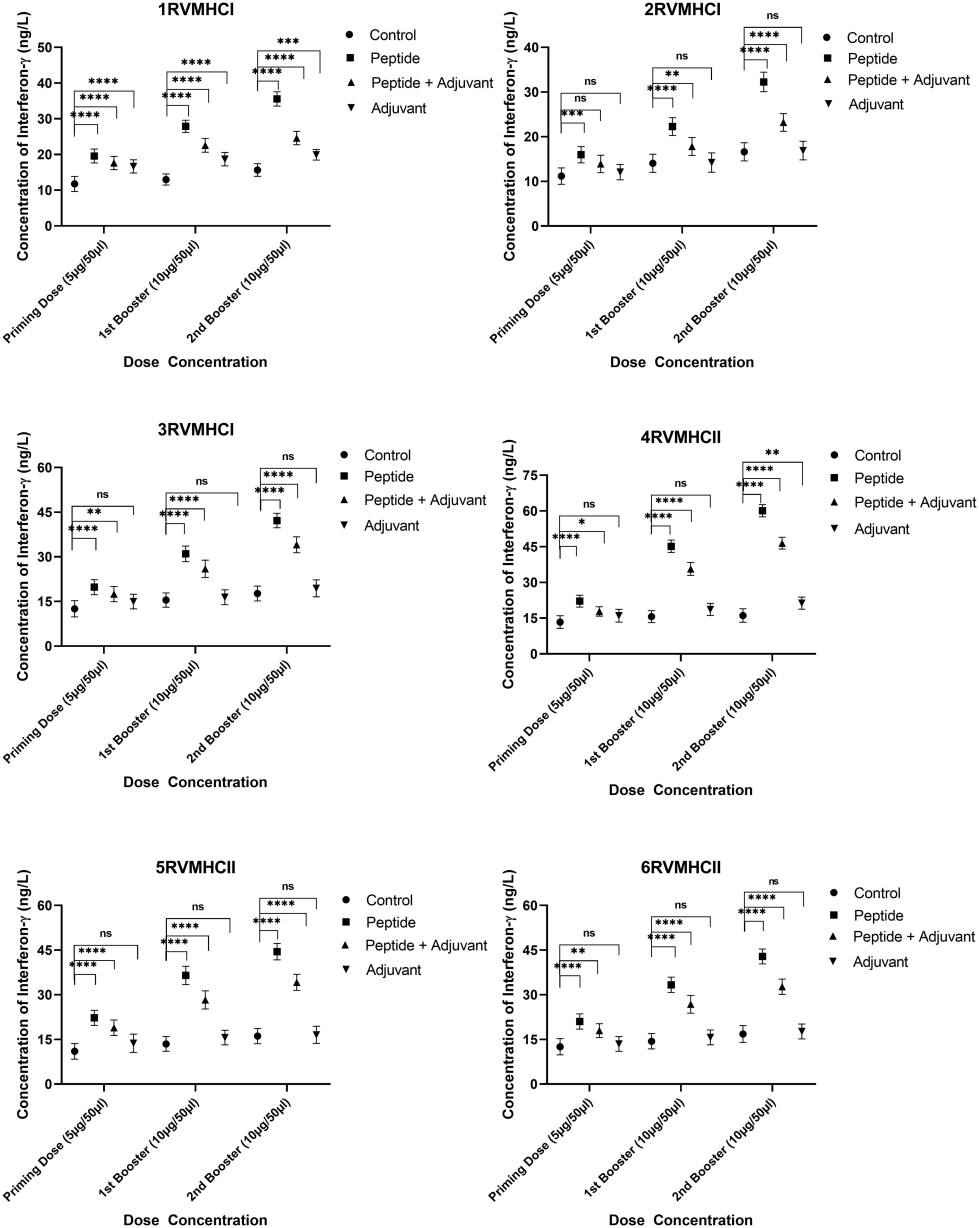
The ELISA kit was used to quantify the IFN-γ level (ng/L) in the sera of mice that received different doses of synthetic peptides, with and without adjuvant. The mean of six mice was represented by error bars. Using GraphPad Prism version 9, two-way ANOVA and Dunnett’s multiple comparisons tests revealed a significant difference (*P* < 0.05). (*p ≤ 0.05 indicates, **p < 0.01, ***p < 0.001,****p < 0.0001), ns: non-significant.

The IFN-γ content in blood sera from mice immunized with CD4^+^- and CD8^+^-specific peptide vaccine candidates were examined. Dunnett’s multiple comparison test with two-way ANOVA was performed to compare IFN-γ concentrations within treated groups with their respective control groups. All peptides produced higher IFN-γ concentrations to develop immunity against RSV, but peptides of CD4^+^ (MHCII) showed significant results compared to CD8^+^ (MHCI) peptides. Compared to the control and adjuvant groups, the second booster dose conferred more substantial immunity in the independent groups of all peptides. At the second booster dose in mice that had received immunization, the 3RVMHCI peptide alone caused a statistically significant increase in IFN-γ (42.19 ng/L) with a *p*-value less than 0.0001 compared to the control group. This was succeeded by the 1RVMHCI peptide (35.57 ng/L) acting independently. 4RVMHCII independently (without adjuvant) induced the highest IFN-γ production among all peptides and other treated groups. At the second booster dose, 4RVMHCII produced the maximum amount of IFN-γ (60.08 ng/L), with *p* < 0.0001. In contrast, the priming and first booster doses produced 22.16 ng/L and 45.1783 ng/L, respectively. Each peptide’s adjuvant group had a negligible increase in IFN-γ compared to controls (**Figure 7**).

#### 3.6.3 Granzyme B assay

**Figure 8:**
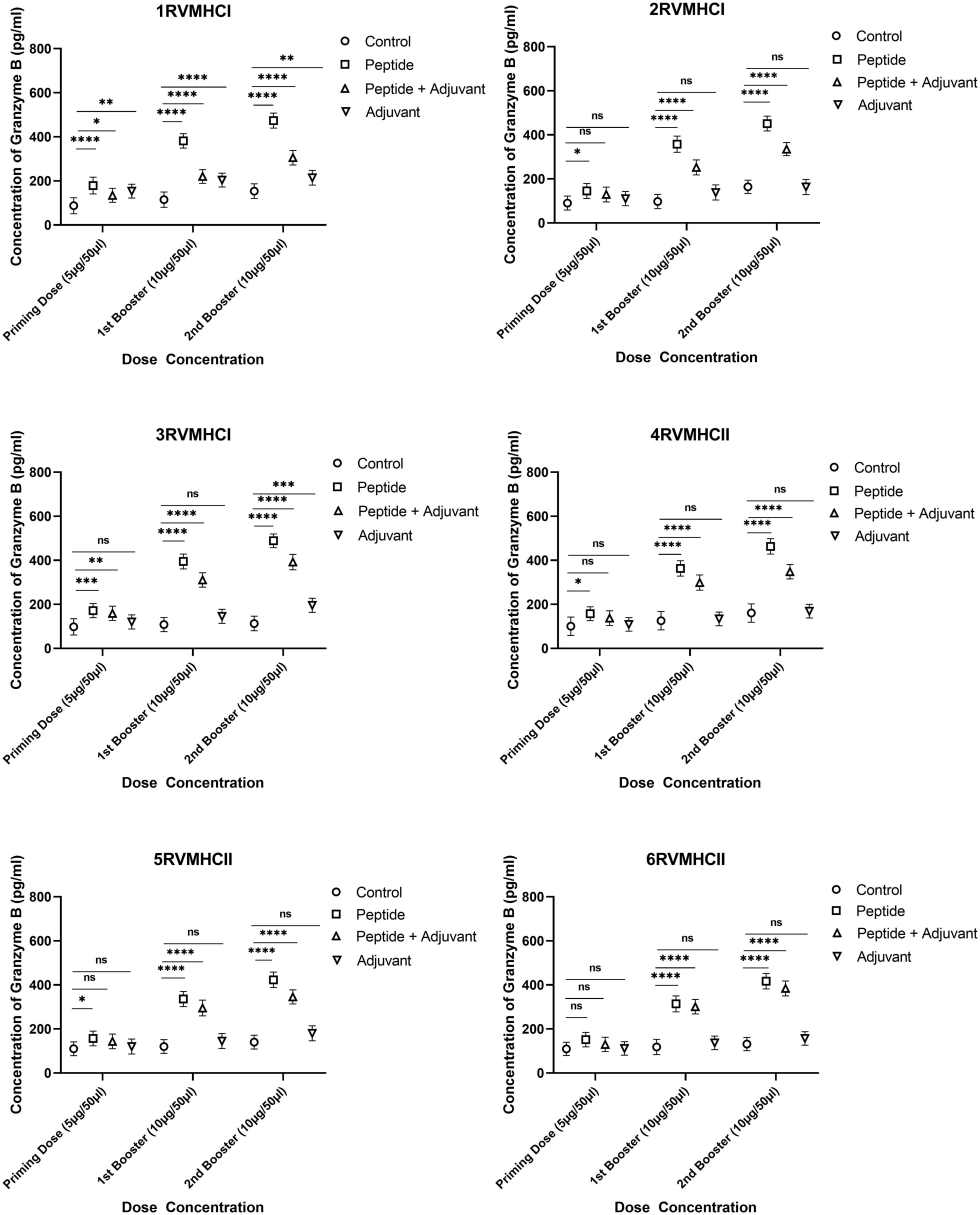
The granzyme B ELISA kit was used to quantify the granzyme level (pg/ml) in the immunized mice’ sera with synthetic peptides at different doses. Error bars showed the mean (SD) from *n* = 6 mice per group. Using GraphPad Prism version 9, two-way ANOVA and Dunnett’s multiple comparisons tests were used to find a significant difference (*P* < 0.05). (*p ≤ 0.05, **p ≤ 0.05, ***p ≤ 0.05, ****p ≤ 0.0001), ns: non-significant.

To calculate the concentration of GzmB molecules, we compared the groups treated with peptides with their respective control groups using Dunnett’s multiple comparison test in a two-way ANOVA. Activated CD8^+^ cytotoxic T cells release the GzmB protein when bound by MHCI molecules. Statistical analysis demonstrated that GzmB proteins were significantly released in response to CD8^+^ peptides compared to CD4^+^ peptides. GzmB levels were lower in mice immunized with peptides along the adjuvant, while only the peptide group showed substantial GzmB release at the second booster dose with a *p*-value of < 0.0001. In total, 3RVMHCI independently induced the GzmB protein at priming, first, and second booster doses of 171.8 pg/ml, 394.77 pg/ml, and 488.68 pg/ml, respectively (**Figure 8**).

### 3.7 Virus neutralization assay

**Figure 9:**
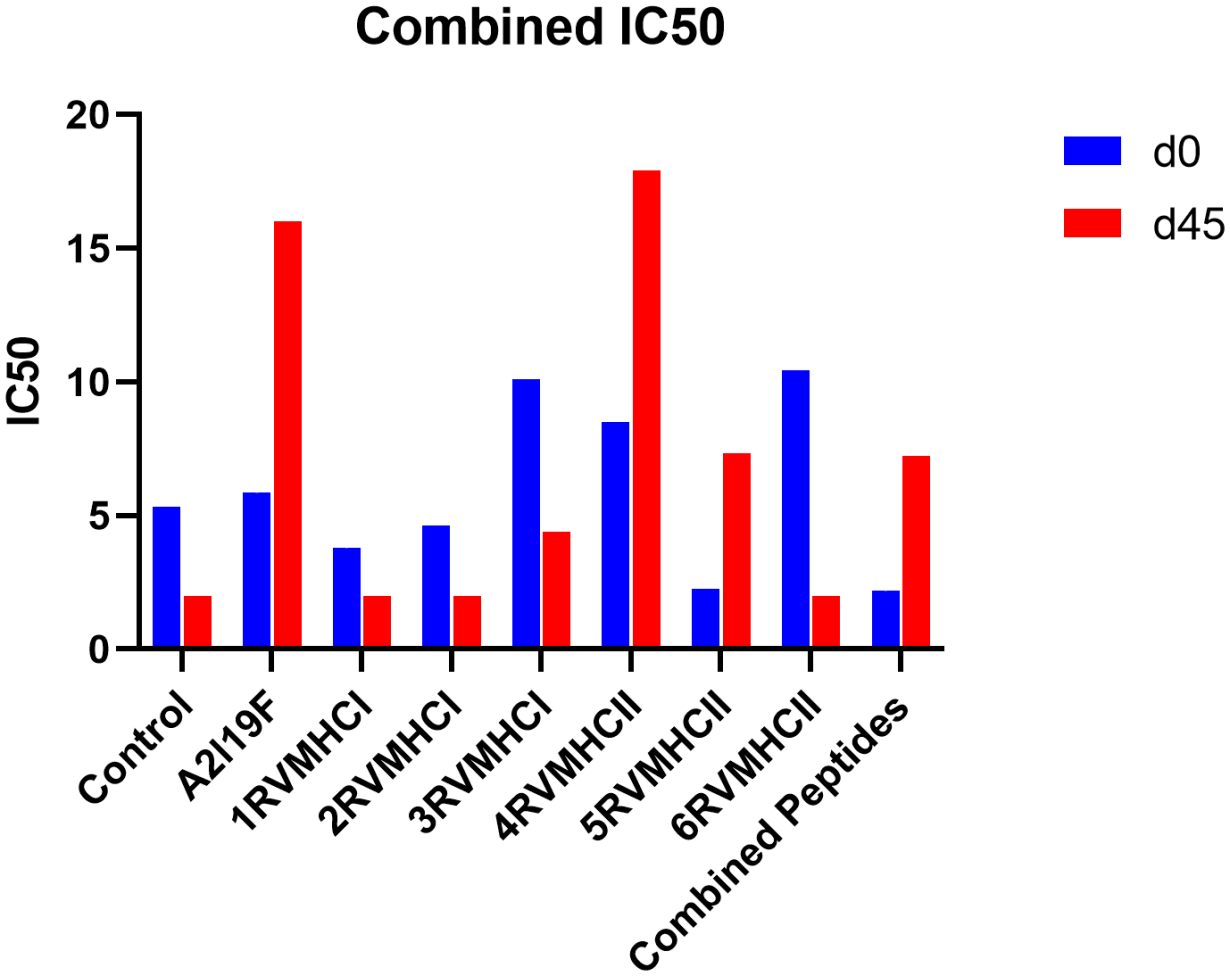
Neutralization of RSV A2l19f virus by sera from pre-infected immunized BALB/c mice on days 0 and 45 pre-infection by FFU count. The assay showed that 4RVMHCII showed greater titer as compared to the control group. A non-linear regression analysis was used to determine the IC50. Using GraphPad Prism version 9, the data showed the mean IC50 from two experimental replicates ± SD.

An approach that is frequently used to quantify the amount of functional antibodies produced in response to peptide injections is the RSV virus neutralization assay. Sera were extracted from immunized mice on days 0 and 45 for neutralization analysis and used diluted in 1:16 ratios. Our results showed that, at day 45, the 4RVMHCII sera had more significant levels of neutralizing antibodies than other peptide groups, although they were comparable to positive control samples (A2l19f) infected with 1 × 10^6^ FFU/ml. The titer of the positive control mice (A2l19f) on day 45 was not fully determined beyond an IC50 of 16. This indicates that the concentration of antibodies needed to inhibit 50% of viral activity exceeded 16 times the initial dilution. The IC50 of 4RVMHCII revealed that a substantially diluted day 45 serum sample (such as a 1:17 dilution) is required to neutralize 50% of the virus. At day 45, 3RVMHCI and 5RVMHCII did not exhibit a significant number of neutralizing antibodies compared to the positive group, nor did they show a substantial increase in antibodies compared to other peptides (**Figure 9**).

### 3.8 Flow cytometry analysis

**Figure 10:**
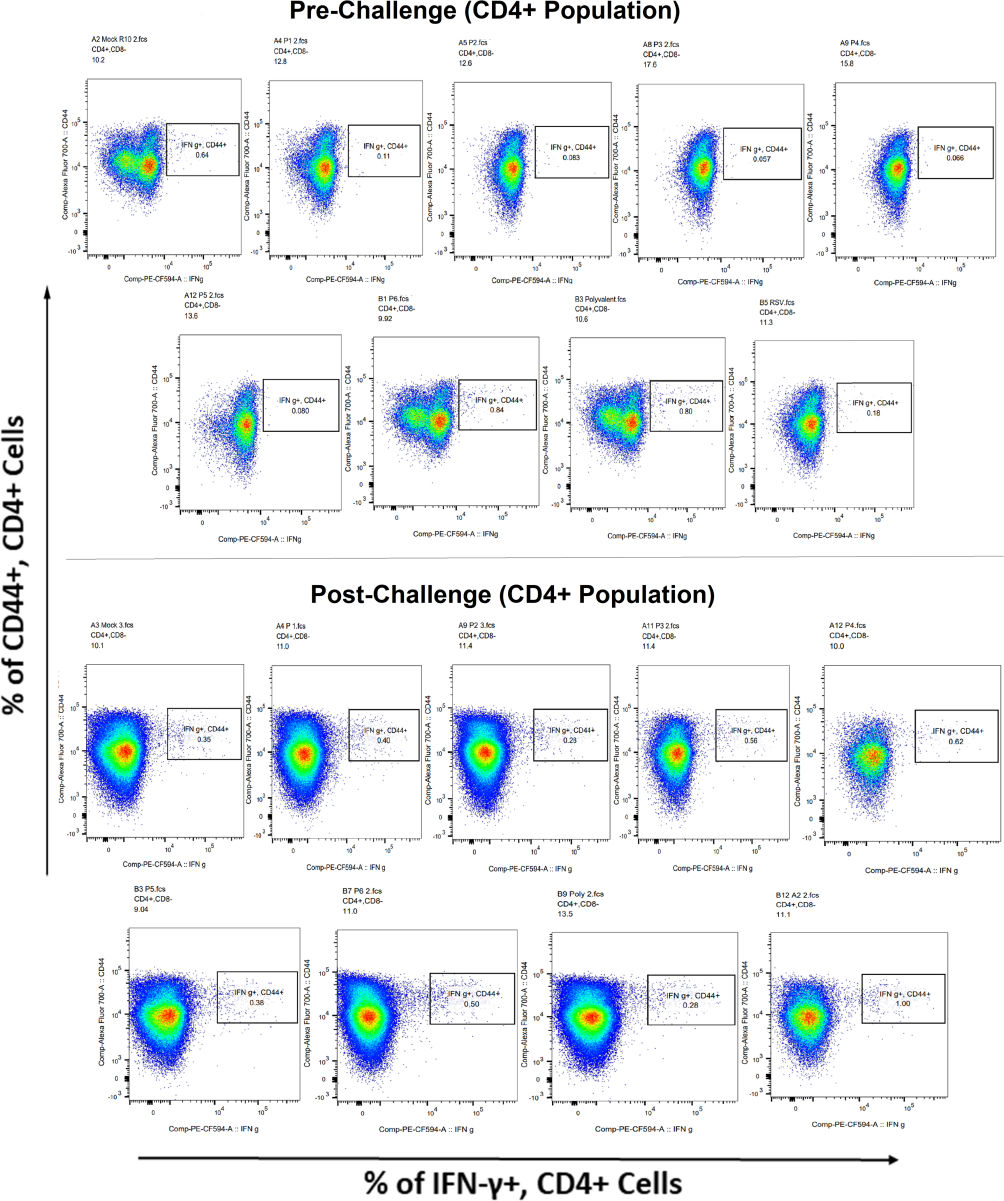
Analysis of the IFN-γ^+^ subpopulation in CD4^+^ and CD3^+^ cells using flow cytometry both before and after they were challenged with the RSV A2l19f strain.

**Figure 11:**
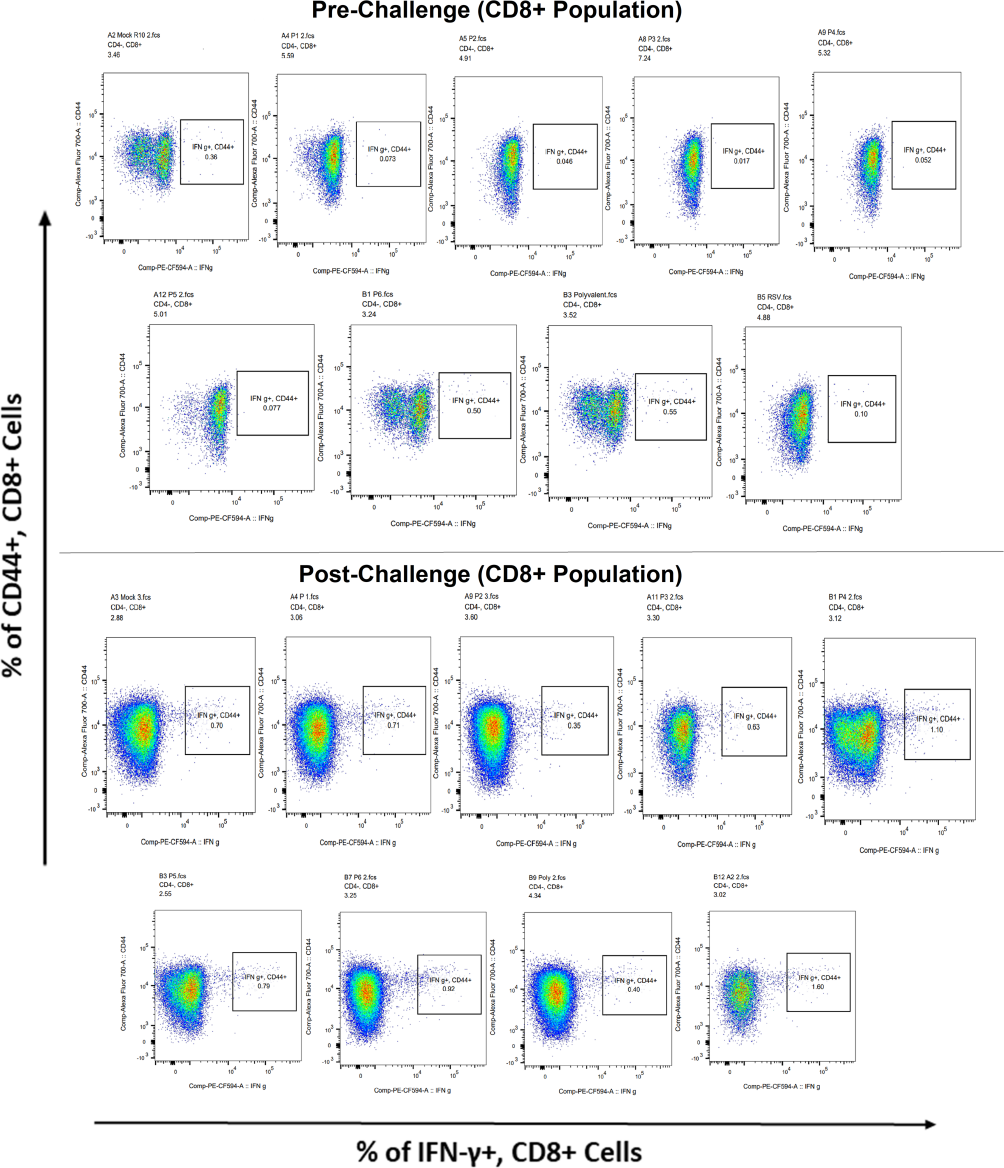
Analysis of the IFN-γ^+^ subpopulation in CD8^+^ and CD3^+^ cells using flow cytometry both before and after they were challenged with the RSV A2l19f strain.

**Figure 12:**
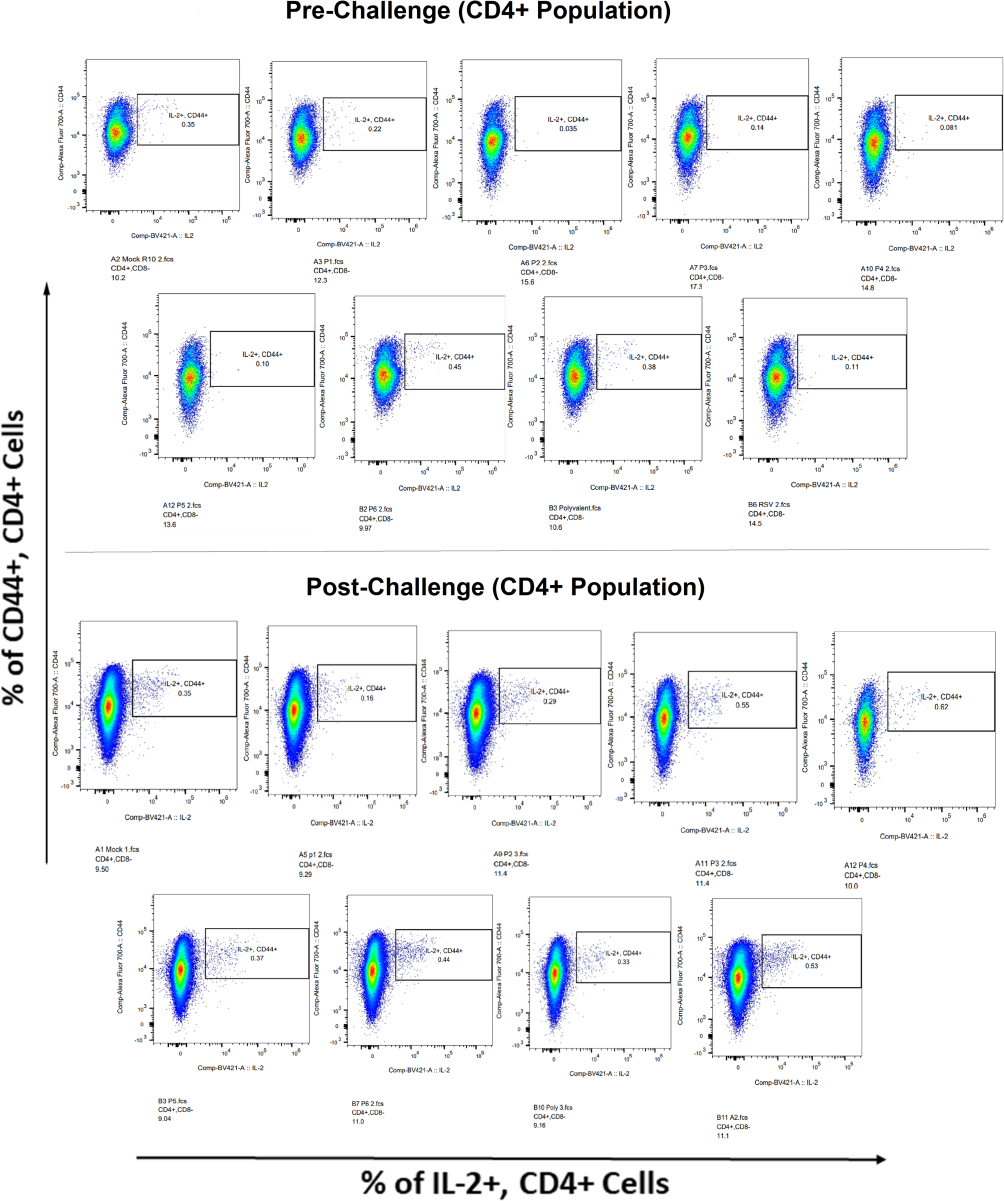
IL-2+ subpopulation in CD4^+^ and CD3^+^ cell populations using flow cytometry analysis both before and after RSV A2l19f strain challenge.

**Figure 13:**
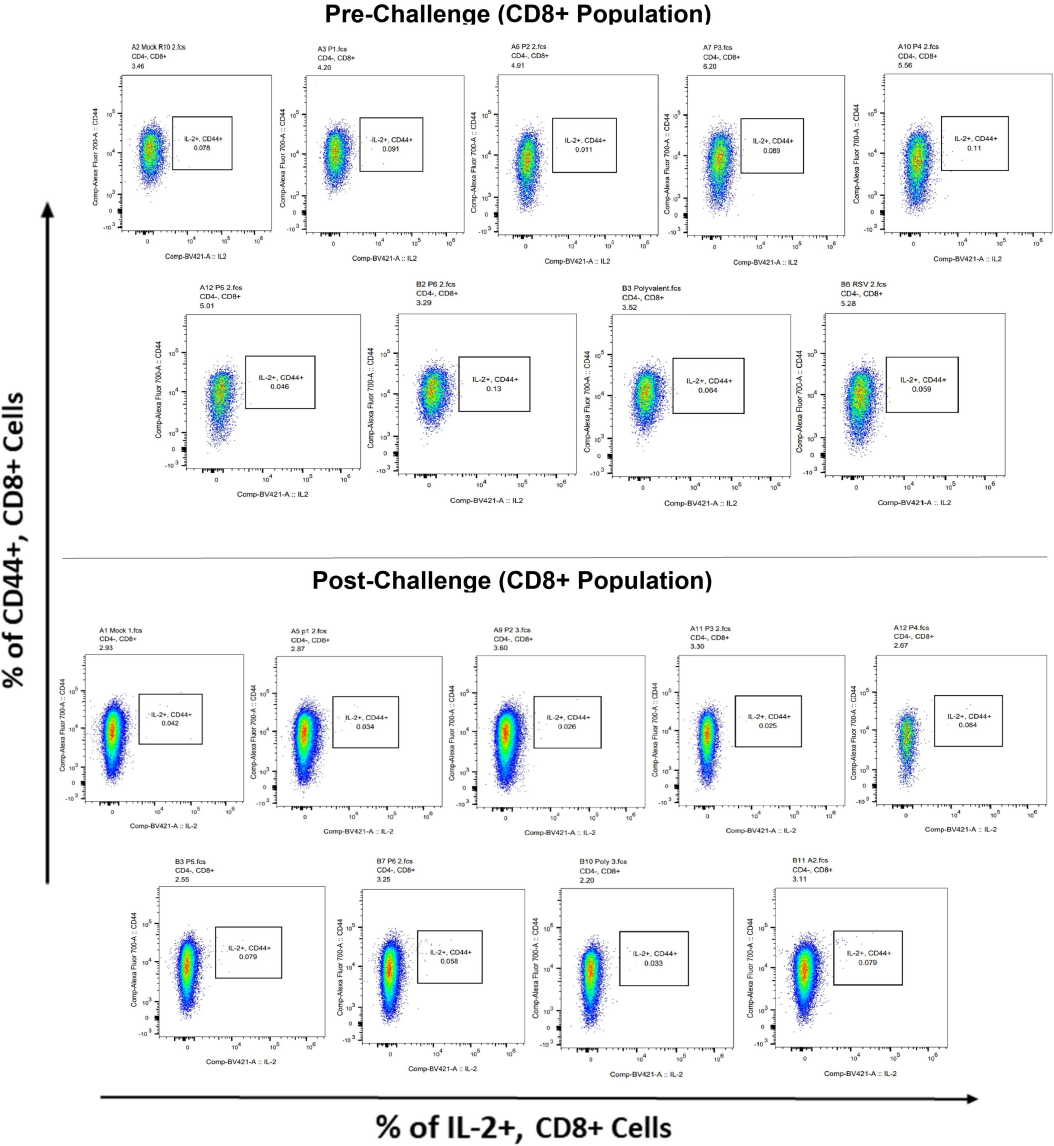
IL-2+ subpopulation in CD8^+^ and CD3^+^ cell populations using flow cytometry analysis both before and after RSV A2l19f strain challenge.

Surface markers and intracellular cytokine labeling were used to assess the immune response to T cells from immunized mice’s splenocytes obtained before and on the fourth day of challenge with A2l19f RSV. The forward scatter plot (FSC) (proportional to size) and side scatter (SSC) (based on cell granularity) were used to gate on lymphocytes, which have low FSC and low SSC. The scatter plot is divided into four quadrants, of which quadrant 4 at the right upper corner shows a double positive population. Before infection, only 6RVMHCII showed high IFN-γ^+^-producing cells compared to the unstimulated group. Although all peptides demonstrated elevated IFN-γ^+^ subpopulations in an unstimulated group before the challenge, 3RVMHCI, and 4RVMHCII showed a substantial increase in IFN-γ^+^ expression in the CD4^+^ population after the challenge relative to the unstimulated group and somewhat similar to the group treated with A2l19f (**Figure 10**). In the case of CD8^+^ pre-infection, the 4RVMHCII, and 6RVMHCII showed increased IFN-γ^+^ subpopulation compared to unstimulated samples, but after challenging, only the 4RVMHCII peptide showed a significant increase in IFN-γ^+^ subpopulation (**Figure 11**). In both CD8^+^ and CD4^+^ populations, the A2l19f RSV-infected group showed greater IFN-γ^+^ expression compared to the groups of mice stimulated with peptides and untreated. Similarly, flow cytometry was used to quantify the IL-2^+^, and CD44^+^ subpopulations among the CD4^+^, and CD3^+^ populations. 6RVMHCII and A2l19f groups showed noticeably higher amounts of IL-2^+^, and CD44^+^ subpopulation expression than their respective unstimulated group and other peptide-stimulated groups before the challenge. However, only peptide-6 statistically showed a significant change. On the other hand, peptides 3–6 showed an elevated subpopulation of CD44^+^ and IL-2^+^ within CD4^+^, and CD3^+^ populations after 4 days of infection (**Figure 12**). However, in the case of CD8^+^, 4RVMHCII peptide substantially increased after the challenge about unstimulated groups. Compared to mice without pre- and post-infection, the A2l19f RSV-infected group exhibited higher IL-2^+^ expression in both CD4^+^ and CD8^+^ cell populations with peptide stimulation. A scatter plot was created with the intracellular marker for IL-2^+^/IFN-γ^+^ on the *x*-axis and the surface markers for CD44^+^ populations on the *y*-axis within CD4^+^/CD8^+^ (**Figure 13**).

### 3.9 Lung virus load

**Figure 14:**
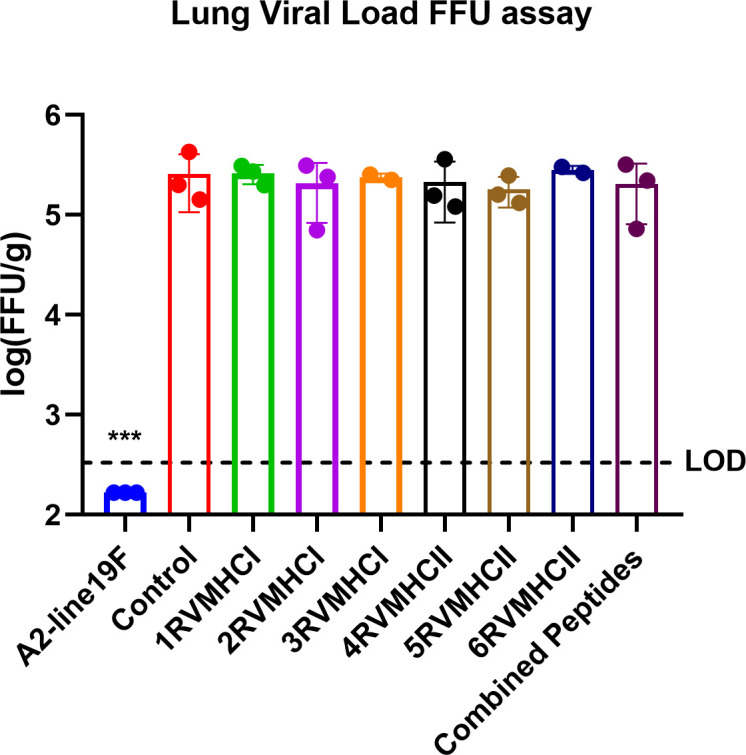
The FFU assay was used to evaluate vaccine attenuation on the fourth day of infection, immunized BALB/c mice were intranasally infected with A2-line19F. Only RSV virus, A2l19f, had a viral load that was lower than the detection value (LOD). Data are mean ± SEM. A significant decrease in lung viral load was indicated by ***. Using GraphPad Prism version 9, a one-way ANOVA with Tukey’s multiple tests was used to evaluate significance (*p* < 0.0001).

The lung was taken from mice immunized with peptides, infected with A2l19f, control group after 4 days of infection before and after challenge, and homogenized cells. FFU results showed a measurable virus load in the mice’s lungs immunized with each of the six peptide groups. Regarding molecular diagnostic assays, LOD is commonly considered to be the lowest amount of target that can be identified in about 95% of repeat measurements. An *in-vivo* investigation revealed that none of those mentioned above peptides decreased the amount of virus in the lungs of immunized mice, since the virus load exceeded the level of LOD and was comparable to that of a control group (**Figure 14**). A2l19f RSV-infected mice showed a non-detectable virus level because RSV-specific antibodies were produced and effectively neutralized the virus upon infection, compared to predicted peptides, which had a geometric mean titer of 5–5.5 log_10_ of FFU/g lung. Although the candidate peptides showed some promising qualities in *in-vitro* studies, however, further animal studies are for validation purposes.

## 4 Discussion

The human respiratory syncytial virus (hRSV) is a serious risk to newborns, young children, and immunocompromised individuals. Including the severity, the virus’s high level of mutability and capacity to inhibit the human immune system make it challenging to develop a licensed vaccine (45). However, recent developments have produced several vaccine candidates with high potential, such as vector-based, live-attenuated, and subunit vaccines. Two subunit candidates, that is, Pfizer’s F protein vaccine “Abrysvo” (46) and GlaxoSmithKline F protein vaccine “Arexvy” (10) have proven successful in clinical trials to effectively reduce RSV-related LRTIs by more than 80% in pregnant women to provide passive immunity to newborns and in elderly people, respectively. The “lollipop-shaped” prefusion protein (preF) of the virus is the target of both RSV vaccines. After fusion with the host cell receptor, this protein transforms into a “crutch-shaped” post-fusion (postF) form resulting in an infection (47). Either the viral and cell membrane merge or another unidentified process causes the highly metastable preF to spontaneously reorganize into the energetically favorable postF conformation, resulting in the acquisition of the postF state. The two varieties are being studied as potential vaccination candidates due to their distinct antigens. Injection site discomfort, fatigue, myalgia, headache, and preterm births are the adverse effects of Arexvy (10). Abrysvo reported that adverse responses in pregnant women were headache, muscle discomfort, injection site pain, nausea, the chance of congenital disabilities, and loss. In contrast, older people showed adverse effects such as muscle pain, injection site pain, exhaustion, and headache (11). RV was used to find putative T-cell epitopes, broadening the pool of possible vaccine candidates. This innovative strategy has the potential for long-term cell-mediated immunity and supports traditional vaccine development techniques. Using various techniques from genomics, proteomics, and bioinformatics, this approach predicts vaccines for a range of infectious diseases. This strategy can potentially design CD4^+^ and CD8^+^ T-cell therapeutic candidates to combat persistent viral infections and develop versatile preventive vaccinations. When paired with adjuvants, epitope-based vaccinations provide a simple manufacturing process and strong immunogenicity (17, 48).

As previously reported, six of the 10 RSV MHC classes I and II epitopes identified by meta-analysis were selected for additional investigation due to their antigenicity, immunogenicity, toxicity, and low-binding energy (23). The successful development of a potent RSV vaccine is a crucial endeavor, with experimental validation *in-vivo* using animal models being of utmost importance (19). In this investigation, healthy female BALB/c mice were used to validate the predicted peptides, showing notable immunological responses, especially for the peptides 3RVMHCI and 4RVMHCII. Hematological tests showed that immunized mice had a higher number of LYM, NEU, and WBC values. ELISA assays further supported immune responses, which revealed elevated IgG antibody concentrations in the treated groups. 4RVMHCII peptide showed substantial antibody production (57.85 mg/ml) compared to other peptides and control with a *p*-value of <0.0001. However, CD4^+^ epitopes were especially important in triggering the production of IFN-γ, which is vital against viral infections. The most significant amount of IFN-γ was generated by the 4RVMHCII, highlighting its potent candidate vaccine. As previously reported, this increase offers encouraging information for developing an effective RSV vaccine (35). CD8^+^ cells provide acquired immunity by binding to MHCI surface molecules on APCs (49). GzmB, which functions through caspase-dependent and independent mechanisms, is a powerful pro-apoptotic enzyme employed by CTLs and NK cells to destroy infected or tumor cells (50). In this study, we found a significant concentration of GzmB in epitopes-treated animals indicating the stimulatory response of CD8^+^ T-lymphocytes.

The importance and efficacy of vaccine candidates against infection were tested by experimental validation in infected animal models. Various cell line tests were also performed such as serum virus neutralization (SVN), flow cytometry, and an estimation of lung virus load. According to the SVN assay using human Hep-2 cell lines, 4RVMHCII revealed significant levels of neutralizing antibodies, similar to the virus A2l19f. With the aid of flow cytometry, numerous cell populations in various tissues can be precisely characterized (51). The results showed that the 4RVMHCII peptide activated IFN-γ^+^ and IL-2^+^ producing subpopulations after exposure to the RSV virus. Despite eliciting immunological responses, none of the peptides neutralized RSV in the lungs of mice, indicating minimal efficacy in lowering viral infectivity. This inefficacy might be explained by certain characteristics such as peptide hydrophobicity, poor solubility, and minimal surface exposure stimulating the immune system. The 3RVMHCI and 4RVMHCII F protein peptides demonstrated some potential to induce an immune response, as reported that the F protein is an essential vaccine candidate due to its increased antigenicity and immunogenicity along with its conservancy across RSV strains (52). In general, the work highlights the importance of future development and testing.

The Pfizer Abrysvo vaccine targets the RSV F protein, a critical factor in viral entry, provides active immunization in pregnant women. Antibodies from vaccinated mothers are passed through the placenta providing passive immunization for infants under six months of age, RSV infection (53). Our peptides cause humoral and cellular reactions, which may provide defense by neutralizing the virus and eradicating infected cells. Targeting both B and T cells may offer a more effective and multifaceted defense against RSV and possibly reduce the vulnerability of the virus to escape. Although the Pfizer vaccine is only recommended for pregnant women between 32 and 36 gestation age (11), our vaccination options target all age groups, thus offering insightful information about possible candidates for vaccine.

In conclusion, this study predicted three CD4^+^ and CD8^+^ peptides from RSV structural proteins including F, G, and SH. In both healthy and diseased mice, it was discovered that two of the F protein epitopes, 3RVMHCI and 4RVHCII, significantly elicited an immune response in a healthy animal model. *In-vitro* studies also showed that 4RVHCII produced peptide-specific antibodies that were effectively neutralized. These findings suggest that these two epitopes showed promising effects as potential vaccine candidates; however, further safety and clinical trials are required.

## Data availability statement

The original contributions presented in the study are included in the article/**Supplementary Material**. Further inquiries can be directed to the corresponding authors.

## Ethics statement

The animal studies were approved by Institute of Molecular Biology and Biotechnology’s Bioethics Committee for Animals under Approval No. IMBB/02/2019. The studies were conducted in accordance with the local legislation and institutional requirements. Written informed consent was obtained from the owners for the participation of their animals in this study.

## Author contributions

SN: Writing – original draft, Methodology. JG: Writing – review & editing, Data curation. SZ: Writing – original draft, Methodology, Investigation. AA: Writing – review & editing, Formal analysis, Conceptualization. LA: Writing – review & editing, Methodology, Formal analysis. CR: Writing – review & editing, Methodology. BB: Writing – review & editing, Formal analysis, Conceptualization. SM: Writing – review & editing, Supervision, Formal analysis, Conceptualization.

## Supplementary material

Supplementary Figure 1 Integrative system-level framework for predicting and designing potential epitopes of respiratory syncytial virus (RSV).

Supplementary Figure 2 Functional annotation of screened RSV protein as protein–protein interaction.

Supplementary Figure 3 Molecular interaction of CD8^+^ T-cell epitopes with target MHCI allele HLA-A*01:01 (PDB ID: 1w72) along with binding energies.

Supplementary Figure 4 Molecular interaction of CD4^+^ T-cell epitopes with target MHCII allele HLA-DRA/DRB1*01:01 (PDB ID: 1BX2) along with binding energies.

Supplementary Figure 5 Molecular interaction of polyvalent construct with targets MHCI, MHCII, and TLR3 molecules along with binding energies.

Supplementary Figure 6 The phylogenetic tree showed the relationship between various RSV strains.
