# Supplementary figures and images for: Experimental trials of predicted CD4+ and CD8+ T-cell epitopes of respiratory syncytial virus
Source: Front Immunol. 2024 Apr 2;15:1349749. doi: 10.3389/fimmu.2024.1349749 (PMC11018974; doi:10.3389/fimmu.2024.1349749)

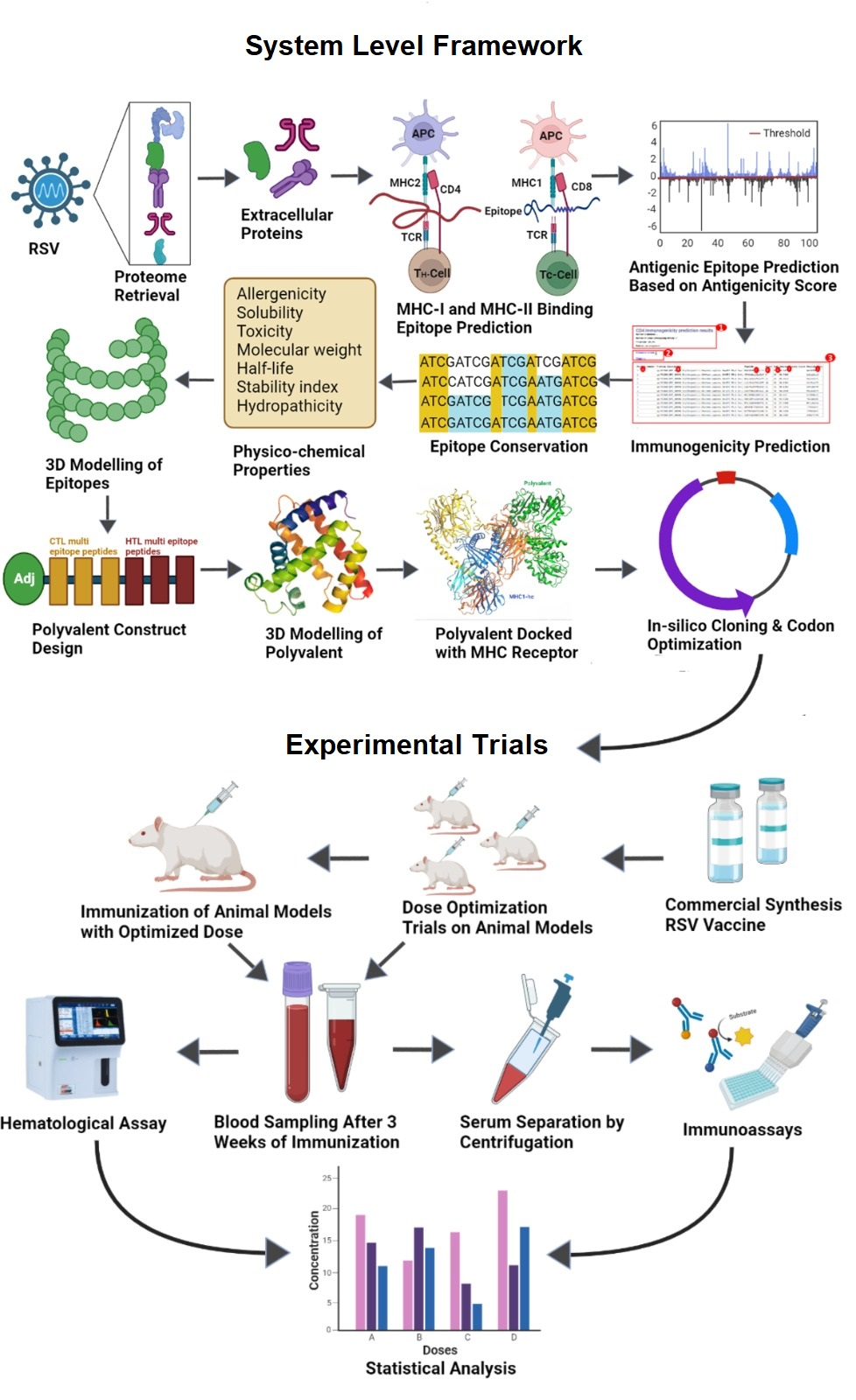

Supplement: Supplementary Figure 1 — Integrative system-level framework for predicting and designing potential epitopes of respiratory syncytial virus (RSV). [file Image_1.tif]

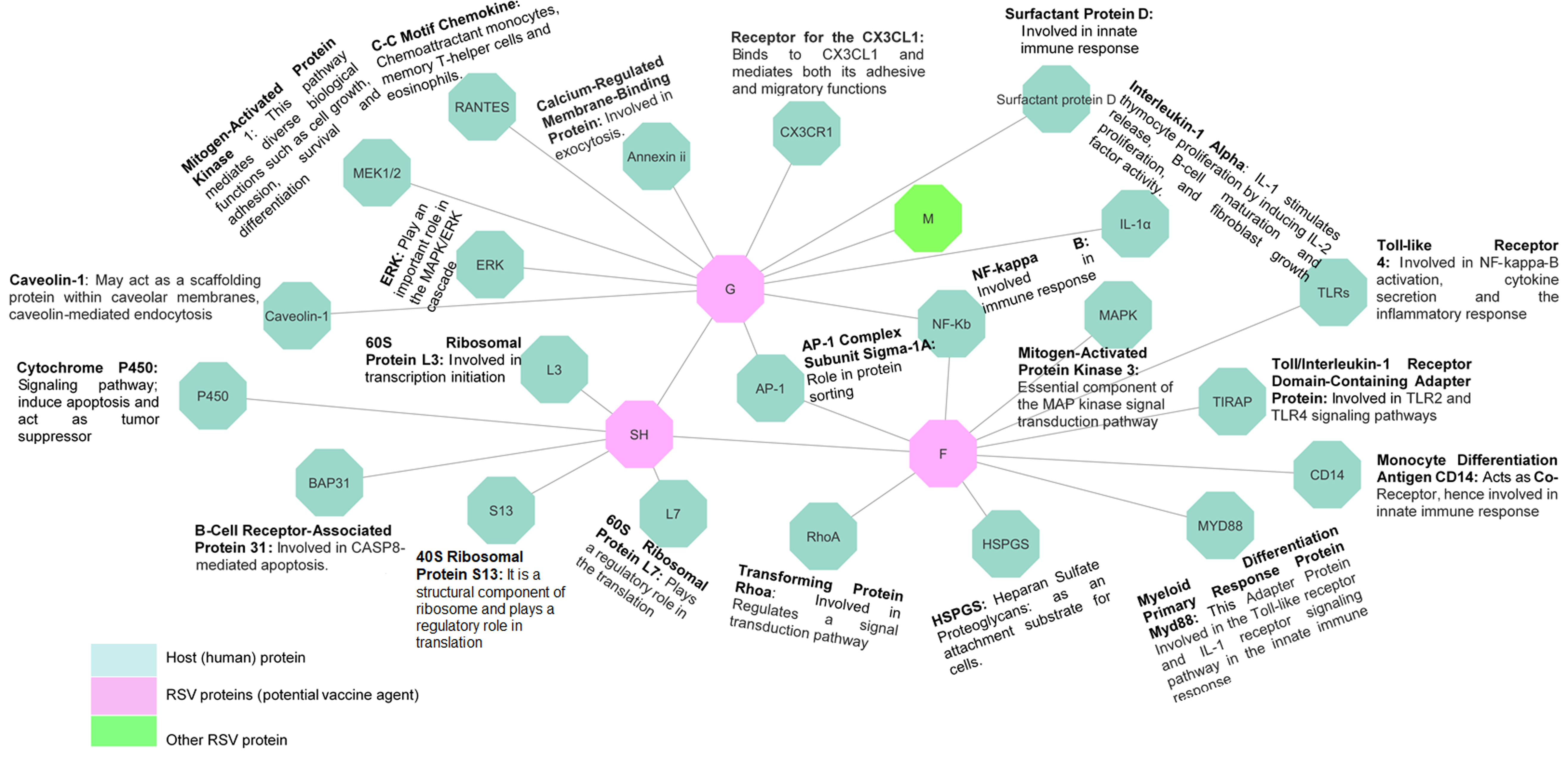

Supplement: Supplementary Figure 2 — Functional annotation of screened RSV protein as protein–protein interaction. [file Image_2.tif]

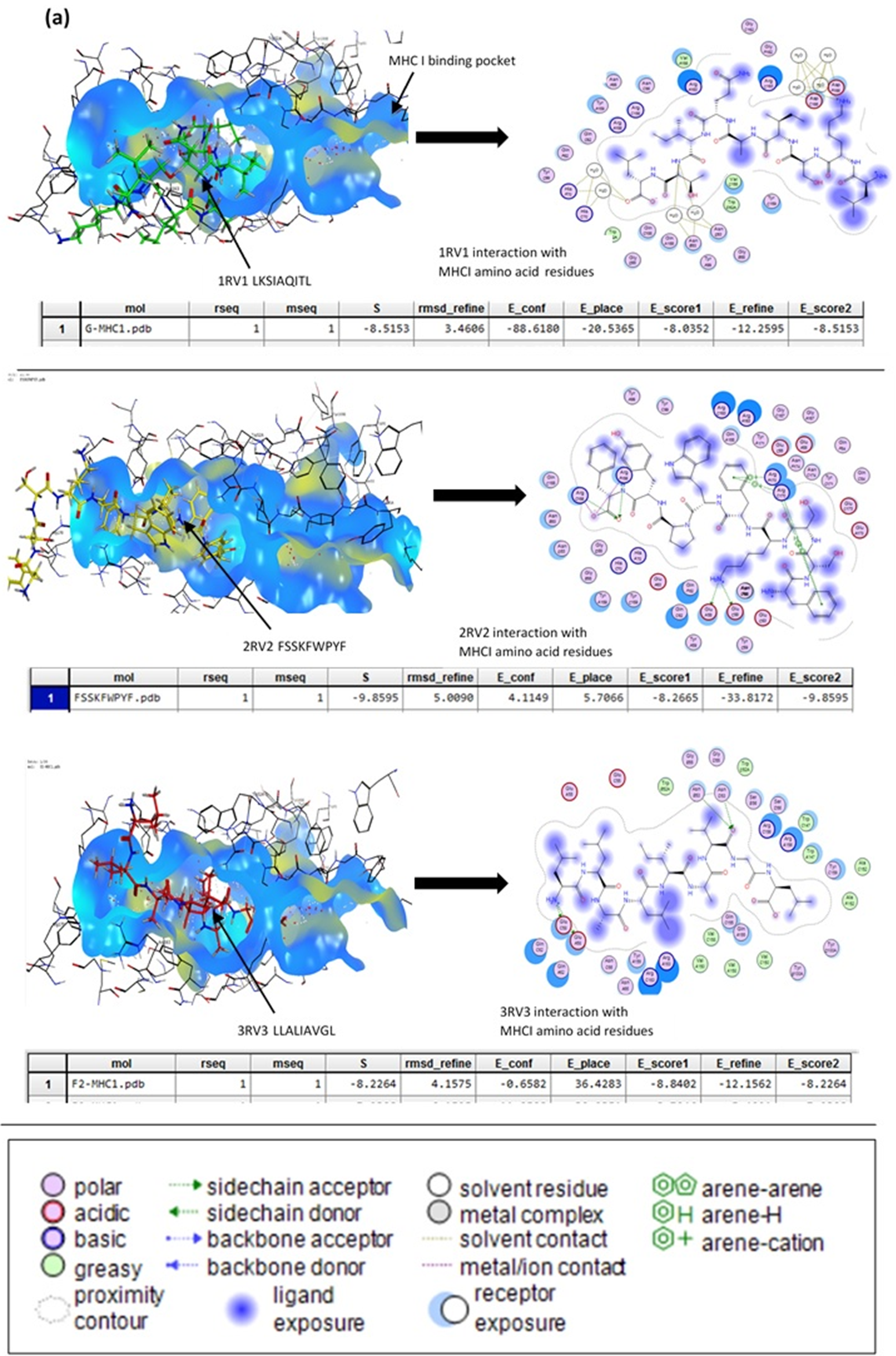

Supplement: Supplementary Figure 3 — Molecular interaction of CD8+ T-cell epitopes with target MHCI allele HLA-A*01:01 (PDB ID: 1w72) along with binding energies. [file Image_3.tif]

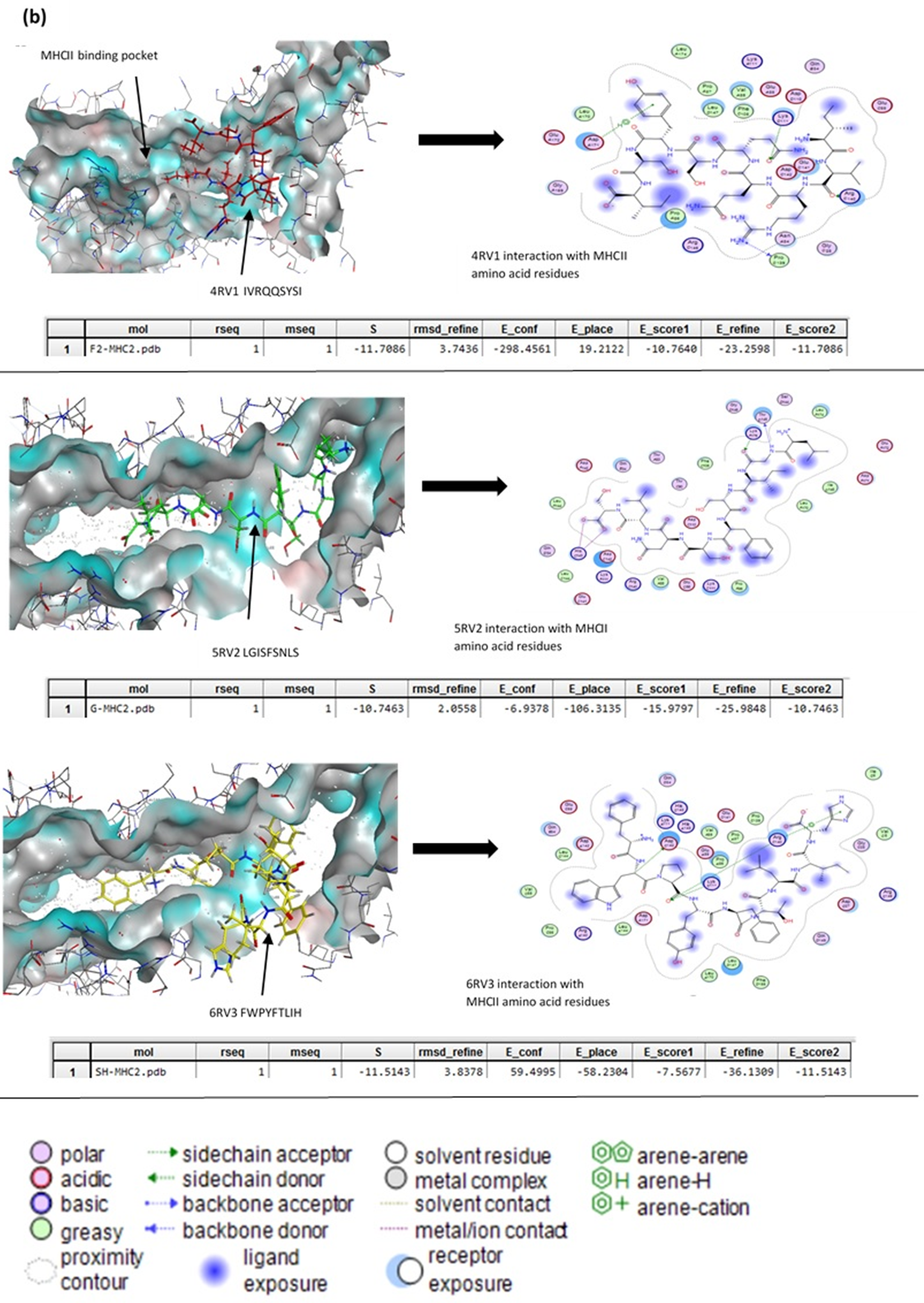

Supplement: Supplementary Figure 4 — Molecular interaction of CD4+ T-cell epitopes with target MHCII allele HLA-DRA/DRB1*01:01 (PDB ID: 1BX2) along with binding energies. [file Image_4.tif]

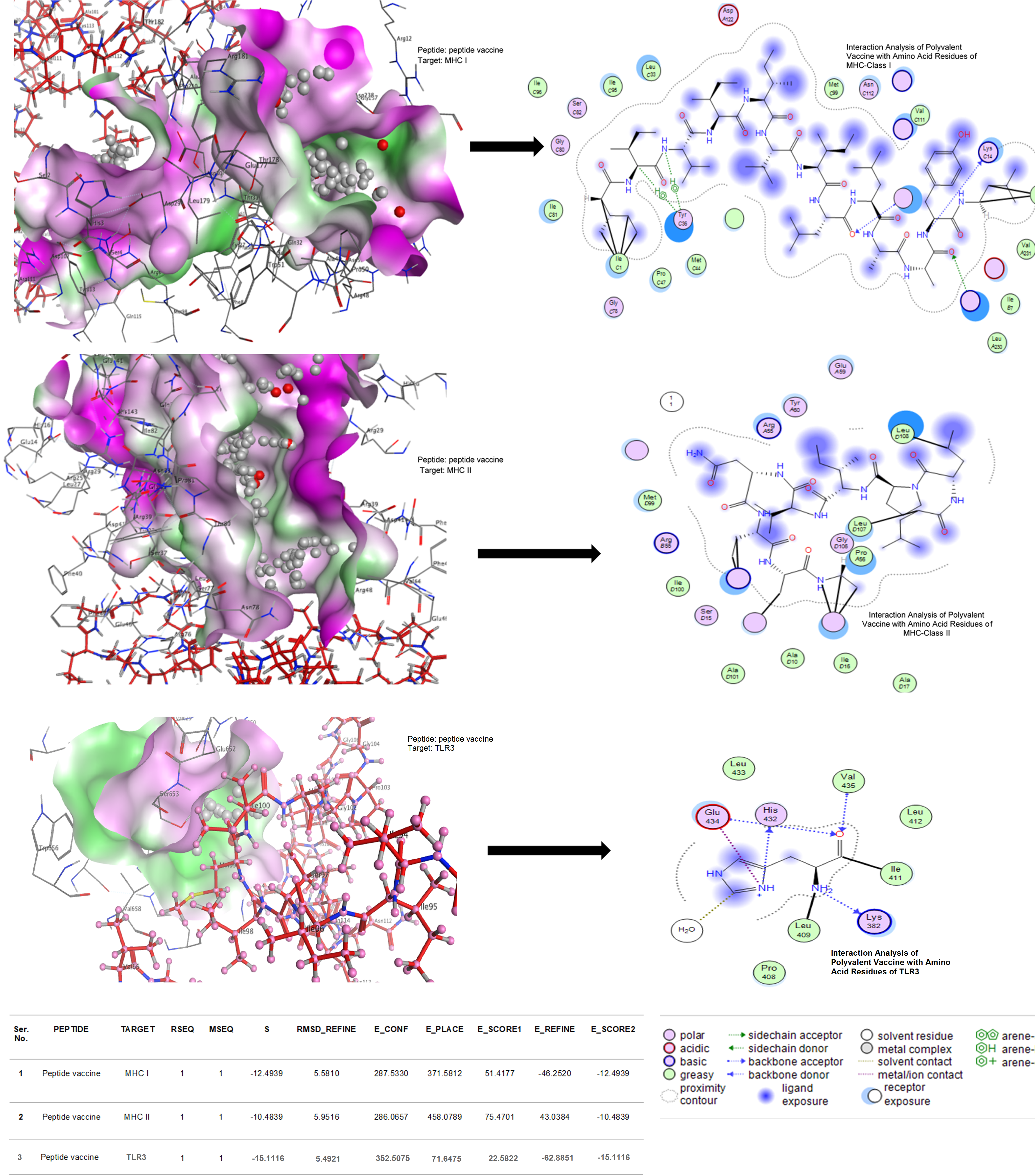

Supplement: Supplementary Figure 5 — Molecular interaction of polyvalent construct with targets MHCI, MHCII, and TLR3 molecules along with binding energies. [file Image_5.tif]

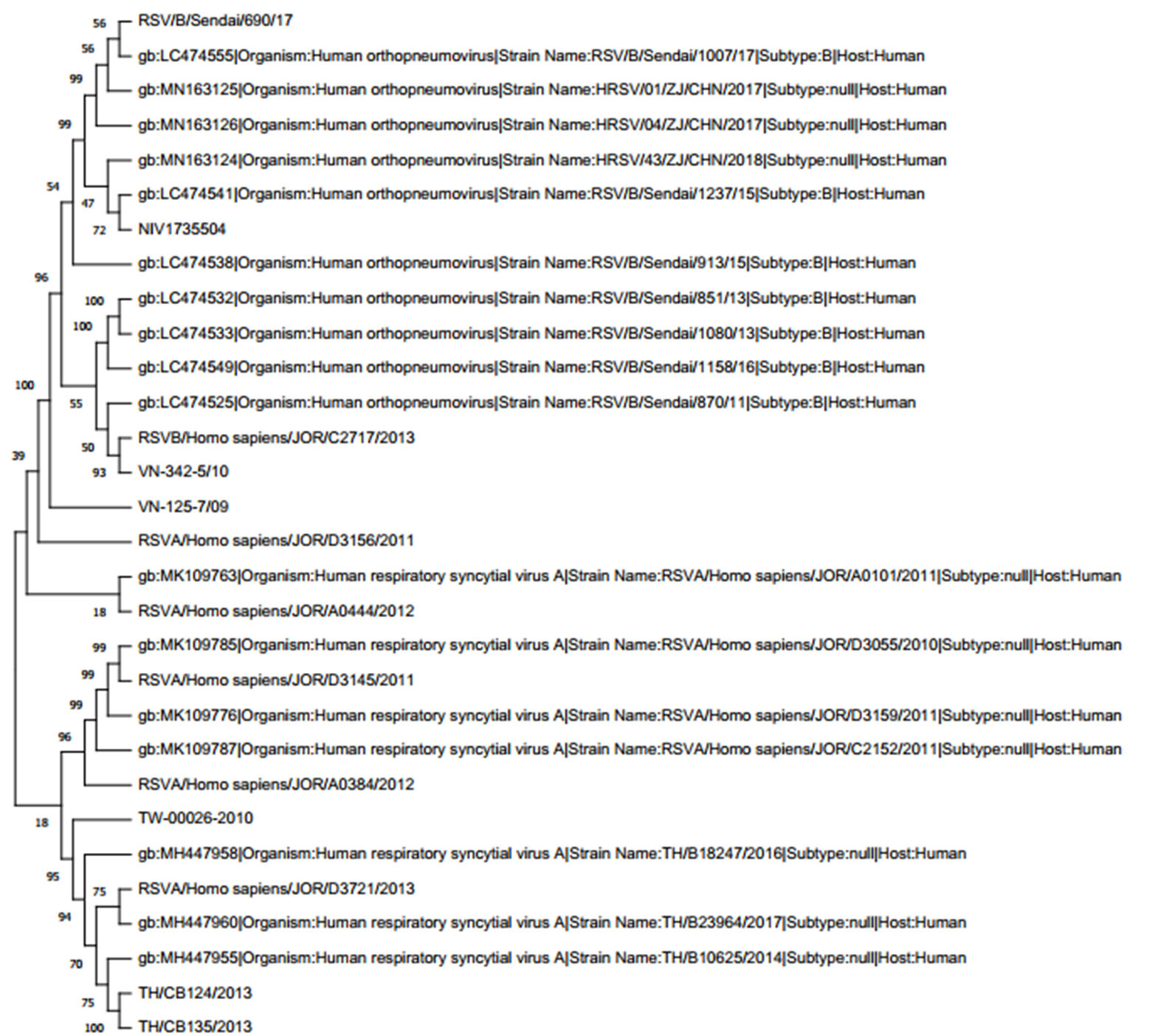

Supplement: Supplementary Figure 6 — The phylogenetic tree showed the relationship between various RSV strains. [file Image_6.tif]
